# Pain Sensitivity and Affective Touch Perception: Evidence from Direct and Vicarious Experiences in Healthy Individuals and Patients with Migraine

**DOI:** 10.3390/brainsci16101009

**Published:** 2026-09-23

**Authors:** Ivana Frigione, Francesca Farinola, Licia Grazzi, Angelo Maravita

**Affiliations:** 1 Department of Psychology, University of Milano-Bicocca, Piazza Ateneo Nuovo 1, 20126 Milan, Italy; ivana.frigione@unimib.it (I.F.); f.farinola@campus.unimib.it (F.F.); 2 Neuroalgology Department, Headache Center, IRCCS Foundation “Carlo Besta” Neurological Institute, Via Celoria 11, 20133 Milan, Italy; licia.grazzi@istituto-besta.it

**Keywords:** affective touch, chronic pain, migraine, allodynia

## Abstract

**Highlights:**

**What are the main findings?**

- Direct and vicarious affective touch induce similar hedonic responses in both healthy and migraine populations.
- There is no evidence that pain influences affective touch perception, which is preserved even in chronic pain. However, chronic pain sufferers may exhibit avoidance behaviours and reduced empathy for others’ pain compared to healthy individuals.

**What are the implications of the main findings?**

- Affective touch-based observation interventions could be applied in populations where physical contact is difficult due to hypersensitivity, allodynia, or in individuals who dislike being touched.
- We establish the importance of personalised approaches that consider both sensory and psychological dimensions of pain experiences.

**Abstract:**

Background/Objectives: As the skin serves as an interface between internal and external environments, pain and affective touch (AT) act as interoceptive and exteroceptive stimuli. Several studies have focused on these stimuli, independently evaluating pain or AT perception in direct or vicarious (observed) stimulation modalities. This study, instead, compared direct and vicarious perception of AT and pain, exploring their potential mutual influence in healthy individuals and patients with a high frequency or history of chronic migraine (CM). Methods: Two identical experiments were conducted with 37 healthy volunteers and 37 CM patients. Participants underwent: (1) a psychometric assessment; (2) a quantitative sensory testing of direct pain thresholds and response to others’ pain (vicarious pain); and (3) response to AT in direct and observed conditions. Results: In both populations, direct and observed AT elicited comparable hedonic responses. Compared to healthy controls, CM patients exhibited significantly higher pain thresholds and reduced vicarious pain sensitivity. No significant mutual influence or correlation was found between pain sensitivity and AT perception in either group. Conclusions: Pain and AT perception show no association, likely reflecting their differing contextual roles: alerting to pain and interpersonal processing for AT. AT perception remains preserved in CM patients, indicating that hedonic touch mechanisms are resilient to chronic pain-induced alterations. The capacity of vicarious touch to evoke affective resonance comparable to direct physical contact might lay the basis for implementing future observation-based interventions for pain management in populations with cutaneous hypersensitivity, allodynia or those who dislike being touched.

## 1. Introduction

The skin, the human body’s largest organ, serves as a crucial interface between the internal and external environments. It serves as a protective barrier against potentially harmful agents and a sensory medium for interaction with the external world through tactile perception. Interoception was initially conceptualised as the sensory processing of signals originating from the viscera, serving primarily reflexive and homeostatic functions and involving signals within the body (e.g., cardiac, respiratory, and digestive). Contemporary perspectives [1] define interoception as “a distributed integrative system” that incorporates information from the viscera and from somatic sources such as the skin (e.g., temperature, itch, pleasure from gentle touch, pain), encompassing vascular, endocrine, and immune systems [2]. Skin-derived signals may simultaneously convey interoceptive and exteroceptive information [3]; thus, they can be acknowledged for their interoceptive significance, as they convey information highly relevant to internal regulation and the preservation of bodily integrity [4]. Skin nociceptive and affective stimuli are transmitted by CT fibres: affective touch and cutaneous pain likewise convey information about the physiological condition of the skin and the body as a whole, consistent with their reconceptualisation as interoceptive stimuli under the revised definition of interoception [5,6,7,8]. Partial overlap between neural mechanisms of pain and CT-optimal touch (affective touch) can explain why affective touch effectively reduces acute pain [9], while no unique trend exists in chronic pain conditions. Affective touch can interact with various chronic pain conditions, including fibromyalgia, musculoskeletal disorders, neuropathic pain, headache, and orofacial pain. It has emerged as a potential non-pharmacological intervention for the management of chronic pain. A brief CT-optimal touch skin stroking resulted in a reduction in self-reported pain severity among individuals with various chronic pain conditions, including primary chronic pain, secondary musculoskeletal pain, and neuropathic pain [10]. Furthermore, affective touch is a recognised factor in attenuating pain responses induced by a temporal summation of second pain protocols, which mimic spinal “wind-up”, the progressive increase in neuronal firing of dorsal horn neurons, usually deemed relevant for the functional evaluation of chronic syndromes. This effect reflects a neural mechanism by which the brain integrates repeated nociceptive input with affective regulatory processes associated with CT fibre activation. This modulation may reflect an interaction between pain processing and emotional regulation, mediated by the affective dimension of CT-optimal stimulation [11]. Notably, CT-optimal touch alleviates symptoms of central and peripheral neuropathic pain, as tactile allodynia [12], a common feature of neuropathic pain, characterised by painful sensations in response to normally innocuous stimuli such as light touch [10]. It is recognised that affective touch and pain perception may reflect a complex interplay of personality traits, past and present experiences, and environmental factors. Both fulfil their role as interoceptive stimuli through bodily interactions that can be directly experienced or observed. In the second modality, empathy operates through an embodied simulation mechanism, in which the brain replicates the observed state, enabling empathic resonance [13,14,15]. Two forms of empathy can be distinguished: an automatic, sensory-level resonance, and another, more complex one, based on affective resonance, which entails genuine emotional sharing [16] and can modulate the observer’s somatomotor responses to the pain of others [17]. Given the influence of emotional factors on touch-induced analgesia, it is also plausible that the empathic disposition of the “toucher” may contribute to the modulation of pain. Furthermore, the magnitude of analgesia and the accuracy of the observer’s empathy may be intertwined in touch-related analgesia [18,19]. Visuotactile mirroring also involves affective touch; observing touch engages neural systems implicated in social and affective processing, with potential implications for pain and empathy. Viewing others being caressed at CT-optimal velocities elicits activity in the posterior insular cortex, a region associated with affective touch and interoceptive integration, similar to when touch is directly experienced [20,21]. Additionally, the correlation between emotional awareness and motor resonance underscores the connection between interoceptive awareness and the processing of vicarious touch experiences [22]. Also, affective touch and its vicarious processing contribute to the social modulation of pain and emotion, in which the attachment style influences the integration of social and somatosensory signals [23,24]. Together, these lines of research support a model in which both direct and observed affective touch engage overlapping brain mechanisms that shape interpersonal experience and potentially modulate pain perception. In conclusion, we can state that direct and vicarious responses to affective touch and pain are not purely automatic or bottom-up; they are also modulated by top-down cognitive processes, such as prior expectations, emotional context, the social or moral evaluation of the target, and the perspective from which the experience is observed.

### Aim of the Research

A substantial body of research has investigated pain and affective touch separately, either in direct experience or in an observed (vicarious) modality. Additionally, previous works have focused on the potential therapeutic effects of affective touch and its regulatory role in pain perception. Nevertheless, only a few studies addressed the opposite relationship, namely the influence of pain on affective touch perception.

The primary objective of the present work is to assess pain and affective touch perception within the same individuals, compare their responses across direct and observed stimulation conditions, and explore the potential mutual influence between these two aspects of sensory experience.

Two main aims of the research are:

- To compare the perception of affective touch and pain in direct versus observed (vicarious) stimulation modalities.
- To evaluate whether individual differences in the perception of direct and observed pain stimulation modulate the perception of direct and observed affective touch stimuli.

The following hypotheses are proposed:

- Affective touch is perceived as significantly more pleasant than neutral (non-affective) touch, in line with the current literature [9,25,26].
- Affective touch is perceived as pleasant, even when observed in others, confirming previous research; however, it is experienced as more pleasant and vivid when experienced directly.
- Individual differences in pain threshold and empathy for others’ pain will significantly modulate the perception of affective touch in sufferers of chronic pain, whether in direct or vicarious modalities.

These findings may have significant implications for clinical practice, offering insights into optimising psychological and physical support strategies used by healthcare professionals and others involved in pain management [27].

The present research project consists of two identical experiments, the first (1) conducted on the general population, the second (2) on chronic pain patients of the Headache Centre, Neuroalgology Department of IRCCS Foundation “Carlo Besta” Neurological Institute of Milan, Italy.

A migraine population was selected as a clinical model because it combines recurrent pain with altered sensory processing, providing a relevant context for investigating the interplay between nociceptive and affective-touch processing [28]. Given the bidirectional relationship between the C-tactile and nociceptive systems, the migraine condition may help elucidate how altered pain processing is associated with changes in the hedonic value of touch [28,29].

Experiments 1 and 2 received ethical approval from the Local Committee (Protocol n. 0415865, 2 October 2024, University of Milano Bicocca Ethical Committee) and were conducted in accordance with the Declaration of Helsinki. All participants provided informed consent before participating in the study. The procedures for both experiments are identical and will be detailed only in the first experiment.

## 2. Experiment 1: Healthy Population

### 2.1. Materials and Methods

#### 2.1.1. Participants

A priori power analyses were conducted using G*Power (version 3.1.9.6) [30] to determine the minimum sample size of thirty-five participants, based on repeated-measures analysis of variance (ANOVA—within factors), with an Effect Size F = 0.25; Alpha Error Level *p* = 0.05; statistical power = 0.80, Actual Power = 0.80, and Correlation Among Measures = 0.25, as suggested for exploratory studies.

Thirty-seven healthy volunteers participated in this experiment (mean age: 26 ± 6 years; age range: 20–51 years; 29 female). All of them self-declared they did not suffer from any of the following diagnosed conditions as exclusion criteria:

Neuropathic pain, chronic migraine, severe and documented medical or psychiatric conditions, epilepsy, sensory abnormalities such as multiple sclerosis and peripheral neuropathy, abnormal cutaneous perception or dermatological disease [31].

#### 2.1.2. Procedure

Upon arrival at the testing room, participants were asked to sit comfortably at a table, 50 cm away from a monitor, and connect to a computer.

Since the perception of stimuli such as pain and affective touch is influenced by an individual’s psychological and emotional state, the participants were asked to complete a psychometric assessment (first phase) (Figure 1a) consisting of self-report questionnaires delivered through Qualtrics software v.2.82.0 (Qualtrics, Provo, UT, USA; founded in 2002):

(i) The Beck Depression Inventory (BDI-I) and State-Trait Anxiety Inventory (STAI Y1,2) to assess depressive and state-trait anxiety symptoms [32,33];
(ii) The Allodynia Symptom Checklist-12 (ASC-12), adapted version, to evaluate the presence of cutaneous allodynia in daily life [34];
(iii) The Social Touch Questionnaire (STQ) to consider the attitude toward social touch beyond the experimental setting [35].

Additionally, demographic information, including age, self-declared gender identity, and the presence of pain conditions, was collected.

Then, participants underwent the pain perception assessment (second phase) to assess vicarious and direct pain perception.

The Vicarious Pain Questionnaire (VPQ) [36,37] (www.youtube.com/channel/UCT8goTgWGRsu14NjVaPCSGw/videos Accessed on 15 September 2026). evaluated the features of bodily pain perception while participants watched videos of mild-pain scenes. They were required to indicate whether they had experienced any bodily pain during the video (yes/no) (variable: VP_N = total number of yes pain responses). In the case of an affirmative response, three additional questions were presented to portray the vicarious pain experience: (1) the perceived intensity of pain (variable: VP_INT), assessed using a 10-point Likert scale (1 = very mild pain, 10 = extremely intense pain); (2) the pain localisation; (3) the selection of adjectives best describing the vicarious pain experience, chosen from a list derived from the McGill Pain Questionnaire [38].

Subsequently, the subjective thresholds of perception for tactile (t) and painful (T) stimuli were determined by administering electrical tactile stimuli of increasing intensity using the Digitimer DS7A stimulator [39], applying two Ag/AgCl electrodes on the ventral side of the left forearm at a distance from the wrist to the elbow equal to half the distance between the tip of the middle finger and the wrist line.

The stimulation intensity was calibrated using an ascending method, starting at 0.5 mA continuous current stimulations and increasing in 0.5 mA steps. Each stimulus was 1 s long, with a 500 µs square pulse delivered at 1 Hz.

Participants had to identify and rate the perceived intensity of each stimulus using an 11-point Numerical Rating Scale (NRS) ranging from 0 to 10. Zero corresponded to a no-pain first tactile sensation perceived (tactile sensation = t), 1 represented the beginning of a painful-feeling stimulus (pain threshold = T), and 10 represented an unbearable pain [40]. The procedure ended when the participants’ rating reached the pain threshold.

The complete procedure was run three times, and the participants’ pain thresholds were calculated as the average of the three tactile and pain threshold intensities.

From this, tactile stimuli were defined as non-unpleasant and non-painful (equal to T × 0.5), as well as unpleasant and painful (equal to T × 1.5, T × 1.6, T × 1.7, T × 1.8, T × 1.9, and T × 2).

For the assessment of tactile perception of neutral and painful stimuli, six stimuli perceived as clearly non-unpleasant (T × 0.5) and six stimuli perceived as unpleasant (ranging from T × 1.5 to T × 2) were delivered by the Digitimer in random order. Firstly, the participants were asked to evaluate the tactile sensation, responding with a dichotomic “yes or no”. Then, participants reported the intensity of the tactile stimulus on an 11-point Numerical Rating Scale (NRS) ranging from 0 (no pain) to 10 (the most intense pain imaginable). If the participant rated tactile pain stimuli below 2, the entire procedure was repeated to better assess and confirm the pain threshold. During threshold assessment, participants listened to white noise through headphones to minimise the Digitimer’s noise on the tactile stimulation (Figure 1b).

The order of the pain assessment sessions was driven by the research aim: we were more interested in evaluating vicarious pain than in direct pain thresholds. Because vicarious pain can be influenced by subjective pain experiences, we decided to evaluate it first to avoid any type of influence of previous pain trials [36]. Also, as the main research aim was to evaluate the influence of pain on affective touch perception, we decided to make the participants undergo the pain evaluation before any affective touch experience.

The tactile affectivity assessment presented the main experimental task (third phase). It consisted of two sessions, direct and vicarious (observed), each comprising 12 10 s tactile stimuli presented in a counterbalanced order [21], aimed at assessing the perception of pleasantness and vividness in the tactile experience.

The 21 cm long stroking area on the skin of the participants’ left forearms was marked with a washable marker for the direct touch session. The whole forearm width minimises habituation and CT fibres fatigue [25,41]. The experimenter was trained to deliver the touch at the exact speeds using a headphone connected to a metronome mobile app [42].

Within each session, the twelve stimuli were delivered, in random order, at different speeds: six CT-optimal (affective touch, AT) comprising three stimuli at 3 cm/s and three stimuli at 6 cm/s, and six not CT-optimal (non-affective touch, NAT) at 18 cm/s and 30 cm/s [9,21,43].

Before undergoing the tactile assessment, each participant, regardless of the assigned session order, underwent 5 s AT (6 cm/s) and NAT (30 cm/s) stimulation to familiarise themselves with the tactile sensations they would experience during the experiment.

In the direct session, the experimenter delivered the touch in the proximal-to-distal direction using her hand at four different speeds: CT-optimal (3 cm/s and 6 cm/s) and non-CT-optimal (18 cm/s and 30 cm/s).

In the vicarious trials, participants were asked to indicate their gender identity preference, which was used to select either a female or male arm video, and watch 10 s videos that replicated the touches they had experienced directly.

Each participant underwent a total of twenty-four trials, divided into two sessions of twelve trials each. The order of the two sessions was randomised across participants. After each trial, participants used an 11-point Likert scale to judge the pleasantness (0 = extremely unpleasant, 10 = extremely pleasant) and vividness (0 = not vivid at all, 10 = extremely vivid) of their experience of direct and vicarious tactile sensations (Figure 1c).

The total duration of the experiment was approximately 60 min.

#### 2.1.3. Design and Data Analysis

##### Demographic and Mood Analysis

Demographic data were used to attest to the sample’s psychometric features: proneness to depressive and anxious mood (BDI-II; STAI-Y1, 2); daily attitude toward social touch and perception of neutral touch as painful (STQ and ASC-12).

##### Pain Perception Analysis

The variable direct pain (DP) represents the mean of the pain responses to the six painful stimuli (ranging from T × 1.5 to T × 2) administered by the Digitimer and evaluated using a Numerical Rating Scale (NRS).

From the vicarious pain assessment, a quantitative measure of vicarious pain (VP_N), equal to the total number of pain responses across 16 videos, and the average pain intensity reported (VP_INT) were estimated [44].

##### Main Analyses: Tactile Affectivity

The tactile affectivity task of the third phase of the experiment had a 4 (Velocity: AT = CT-optimal 3 and 6 cm/s vs. NAT CT-non-optimal 18 and 30 cm/s) × 2 (Session: Direct vs. Vicarious) factorial design. The dependent variables evaluated were the pleasantness and vividness of the touch.

Participants had to rate the pleasantness and vividness of tactile stimuli presented in both direct and vicarious sessions. The first two repeated-measures analyses of variance (ANOVAs) were conducted to assess the perceived pleasantness of AT and NAT velocities in the direct and vicarious modalities, exploring each condition and comparing the results with the current literature.

Then, the pleasantness and the vividness scores of the two AT (3 and 6 cm/s) and the two NAT (18 and 30 cm/s) velocities were averaged to obtain one value of pleasantness or vividness for AT/CT-optimal and one value for NAT/CT-non-optimal touch, which were then used to run RM ANCOVA, adding direct and observed pain perception variables as covariates. The Huynh–Feldt correction was applied when the assumption of sphericity was violated. Bonferroni correction for the pairwise comparisons was used as required.

A correlation matrix was used to deepen the relationships among the individual features, pain and AT perception. The Holm correction for multiple correlations was applied.

#### 2.1.4. Results

Thirty-seven participants completed all the questionnaires, and the datasets had no missing values.

Allodynia Symptom Checklist-12 (ASC-12): (M = 1.41, SD = 2.37, range 0–10). Ten participants (27%) scored above the clinical threshold (≥3), reporting symptoms compatible with mild-to-moderate forms of allodynia, even in the absence of a diagnosis of a pain condition. These unexpected findings suggest that somatosensory sensitisation may exist even within non-clinical populations [45]. See the Supplementary Material for all detailed results (Text S1: EXPERIMENT 1: Data Analysis Supplemental Data). 

##### Tactile Affectivity

Analyses were performed using Jamovi software (version 2.3.28). First, the AT and NAT pleasantness and vividness mean intensity ratings were tested for normal distribution using the Shapiro–Wilk test. The test reported some significance for the variables (Vicarious NAT Vividness *p* = 0.022; AT vicarious pleasantness *p* = 0.041). According to Norman [46], a parametric test (ANOVA) can be used to analyse Likert-scale data, although it violates the assumption of normality.

##### Pleasantness

A repeated-measures ANOVA (ANOVA RM) evaluated the effects of touch conditions [*Session*: 2 (Direct vs. Vicarious) and *Velocity*: 4 (AT = 3 and 6 cm/s; NAT = 18 and 30 cm/s)] on perceived pleasantness.

A main effect of *Velocity* [F(1.39, 50.21) = 57.91, *p* < 0.001, η^2^_p_ = 0.617] was found. A main effect of *Session* was not found [F(1, 36) = 2.67, *p* = 0.11, η^2^_p_ = 0.069].

The interaction for Session*Velocity was significant [F(2.76, 99.21) = 9.04, *p* < 0.001, η^2^_p_ = 0.201].

Bonferroni post hoc test comparisons between *velocities* revealed that slow AT velocities (3–6 cm/s) were significantly more pleasant than the faster NAT velocities (18–30 cm/s).

Following the significant interaction for Session*Velocity, the Bonferroni post hoc test revealed that perceived pleasantness in direct and vicarious modalities decreased progressively with the increase in velocity. AT 3 cm/s was rated as the most pleasant compared to direct and vicarious NAT 18 cm/s, and to direct and vicarious NAT 30 cm/s (all *p* < 0.001).

A similar pattern defines the other CT-optimal velocity, AT 6 cm/s, compared to direct and vicarious NAT 18 cm/s, and to NAT 30 cm/s (all *p* < 0.001).

Notably, there was no significant difference between AT 3 cm/s and AT 6 cm/s in direct and vicarious sessions (*p* = 1.00), highlighting a uniformity of pleasantness perception of 3 cm/s and 6 cm/s CT-optimal velocities, also comparable in the actual and observed mode.

The administration modality affects the qualitative perception of CT-non-optimal velocity stimulations, specifically NAT 18 cm/s and NAT 30 cm/s. NAT 18 cm/s was perceived as more pleasant in the direct condition than the vicarious sessions (*p* = 0.003), and there was a similar pattern for NAT 30 cm/s (*p* = 0.01). These findings suggest that, overall, watching NAT is assumed to be less pleasant than when it is experienced on one’s skin. These data indicate that stroking velocity and the administration modality significantly modulate the perception of touch pleasantness. Slow AT velocities were consistently rated as more pleasant across both modalities. On the contrary, NAT velocities showed a different impact on pleasantness depending on the session: the direct touch maintained a relatively constant level of pleasantness (see Figure 2).

##### Pleasantness and Pain Perception

Three repeated-measures ANCOVAs (RM ANCOVAs) evaluated the probability of the influence of direct or vicarious pain perception on the pleasantness. As the AT pleasantness perception is the main object of the analysis, and the two CT-optimal velocities, 3 cm/s and 6 cm/s, showed significant similarities, for the following analyses we considered the overall AT (mean of 3 cm/s and 6 cm/s velocities) and NAT (mean of 18 cm/s and 30 cm/s velocities) tactile experience.

An RM ANCOVA within-subjects [*Session*: 2 (Direct vs. Vicarious) and *Velocity*: 2 (AT; NAT)] was conducted, adding VP Intensity (VP_INT), representing a measure of empathy to others’ pain perception, as the continuous covariate to evaluate the effects on perceived pleasantness.

A main effect of *Velocity* was found [F(1, 35) = 14.86, *p* < 0.001, η^2^_p_ = 0.298]. Bonferroni post hoc test comparison revealed that AT (M = 5.85, SE = 0.32) was significantly perceived as more pleasant than NAT (M = 3.24, SE = 0.24), [mean difference = 2.61, SE = 0.31, (t(35) = 8.28, *p* < 0.001)].

A main effect of *Session* was not found [F(1, 35) = 1.97, *p* = 0.169, η^2^_p_ = 0.053].

A significant interaction for Session*Velocity emerged [F(1, 35) = 15.90, *p* < 0.001, η^2^_p_ = 0.312] (see Figure 3).

The Bonferroni post hoc test confirmed that pleasantness in both direct and vicarious modalities decreased progressively with an increase in velocity.

The direct AT (M = 5.75, SE = 0.33) and vicarious AT (M = 5.96, SE = 0.35) were rated more pleasant than direct NAT (M = 3.62, SE = 0.28) and vicarious NAT (M = 2.86, SE = 0.22).

These data support the superiority of AT pleasantness, which also does not differ between direct and vicarious administration.

An RM ANCOVA within-subjects [*Session*: 2 (Direct vs. Vicarious) and *Velocity*: 2 (AT; NAT)] was conducted, adding VP_N representing a measure of empathy to others’ pain perception, specifically the number of times participants perceived bodily pain while watching the VPQ videos as the continuous covariate, to evaluate the effects on perceived pleasantness.

A main effect of *Velocity* was found [F(1, 35) = 11.44, *p* = 0.002, η^2^_p_ = 0.246]. The Bonferroni post hoc test revealed that AT was significantly perceived as more pleasant than NAT (*p* < 0.001).

A significant interaction for Session*Velocity emerged [F(1, 35) = 12.67, *p* = 0.001, η^2^_p_ = 0.266], consistent with the previous RM ANCOVA results.

These RM ANCOVA results suggest that individual sensitivity to vicarious pain perception did not influence tactile pleasantness.

A last RM ANCOVA within-subjects [*Session*: 2 (Direct vs. Vicarious) and *Velocity*: 2 (AT; NAT)] was conducted, adding direct pain perception (DP) as the continuous covariate to evaluate the effects on perceived vividness.

No main effect of *Velocity* or *Session* or interaction effect for Session*Velocity was found (all *p* > 0.061), indicating that velocity had a comparable effect on pleasantness across the two delivery modes. Also, no significant interaction effect between *DP* and *Session* or *Velocity* or among *DP* * Session*Velocity (all *p* > 0.206) was found.

These results suggest that individual threshold sensitivity to direct painful stimuli does not affect the perception of tactile pleasantness.

##### Vividness

A repeated-measures ANOVA (ANOVA RM) evaluated the effects of touch condition [*Session*: 2 (Direct vs. Vicarious) and *Velocity*: 4 (AT = 3 and 6 cm/s; NAT = 18 and 30 cm/s)] on perceived vividness. Huynh–Feldt corrections were applied because of the violated assumptions of sphericity (Mauchly’s test: *Velocity* factor *p* < 0.001; “Session*Velocity” *p* < 0.001).

A main effect of *Session* emerged [F(1, 36) = 29.92, *p* < 0.001, η^2^_p_ = 0.454].

A main effect of *Velocity* was not found [F(1.36, 48.95) = 0.948, *p* = 0.363, η^2^_p_ = 0.026].

The interaction for Session*Velocity was significant [F(1.97, 70.79) = 3.31, *p* = 0.043, η^2^_p_ = 0.084] (See Figure 4).

Bonferroni post hoc test comparisons between *sessions* showed that the direct touch condition (M = 6.64, SE = 0.33) was significantly more vivid than the vicarious condition (M = 4.61, SE = 0.32) (*p* < 0.001).

Following the significant interaction for Session*Velocity, it was suggested that perceived vividness increases progressively with increasing velocity. The direct NAT at 30 cm/s was rated as the most vivid touch. Otherwise, the Bonferroni post hoc test revealed no significant difference in vividness among all the velocities within the observed or direct sessions (all *p* > 0.05).

Significant differences were found comparing direct and vicarious modalities. The direct NAT 30 cm/s was rated as the most vivid touch compared to all other AT and NAT vicarious ones (all *p* < 0.001). Also, direct NAT touches at 30 cm/s (all *p* < 0.001) and 18 cm/s (all *p* ≤ 0.004) were rated more vivid than their corresponding vicarious ones.

An analogous tendency was observed for direct AT 6 cm/s, resulting in more vivid vicarious ones than AT and NAT touches (all *p* ≤ 0.005). Similarly, AT 3 cm/s was considered more vivid than other AT and NAT vicarious conditions (*p* ≤ 0.033), with the exception of NAT 30 cm/s (*p* = 0.098).

In summary, a significant difference in perceived vividness is noted between direct and observed touch. Furthermore, any touch is experienced more vividly when it involves direct skin contact rather than being observed on video, calling for a central role of skin in social touch.

##### Vividness and Pain Perception

Three repeated-measures ANCOVAs (RM ANCOVAs) assessed the effect of direct or vicarious pain perception on the vividness of AT (mean of 3 cm/s and 6 cm/s velocities) and NAT (mean of 18 cm/s and 30 cm/s velocities) tactile experiences.

An RM ANCOVA within-subjects [*Session*: 2 (Direct vs. Vicarious) and *Velocity*: 2 (AT; NAT)] was conducted, adding VP Intensity (VP_INT), the level of response to others’ pain (i.e., vicarious pain), as the continuous covariate to evaluate the effects on perceived vividness.

A main effect of *Velocity* was found [F(1, 35) = 4.51, *p* = 0.041, η^2^_p_ = 0.114], but Bonferroni post hoc test comparison did not reveal a significant difference in vividness between AT (M = 5.54, SE = 0.28) and NAT (M = 5.72, SE = 0.31), (*p* = 0.480).

A main effect of *Session* emerged [F(1, 35) = 15.43, *p* < 0.001, η^2^_p_ = 0.306]. Bonferroni post hoc test comparison shows that direct touch (M = 6.64, SE = 0.33) is more vivid than vicarious (M = 4.61, SE = 0.32), (*p* < 0.001)] (see Figure 5).

A significant interaction for Velocity* *VP_INT* emerged [F(1, 35) = 4.40, *p* = 0.043, η^2^_p_ = 0.112].

An RM ANCOVA within-subjects [*Session*: 2 (Direct vs. Vicarious) and *Velocity*: 2 (AT; NAT)] was conducted, adding VP_N, which represents the number of times participants perceived bodily pain in vicarious pain experiences.

A main effect of *Session* emerged [F(1, 35) = 16.54, *p* < 0.001, η^2^_p_ = 0.321]. The Bonferroni post hoc test confirmed that direct touch is more vivid than vicarious touch (*p* < 0.001).

Finally, direct pain perception (DP) was added as the covariate in the same RM ANCOVA model.

A main effect of *Session* emerged [F(1, 35) = 14.83, *p* < 0.001, η^2^_p_ = 0.298]. Bonferroni post hoc test comparison confirmed that direct touch is more vivid than vicarious (*p* < 0.001).

##### Pleasantness, Vividness and Cutaneous Allodynia

The unexpected presence of eleven participants presenting mild-to-moderate cutaneous allodynia led us to investigate its role in the pleasantness and vividness experienced with touch. Therefore, the following are exploratory/post hoc analyses.

An RM ANCOVA within-subjects [*Session*: 2 (Direct vs. Vicarious) and *Velocity*: 2 (AT; NAT)] was conducted, adding ASC-12 in daily life (ASC-Daily) as a measure of cutaneous allodynia as the continuous covariate to evaluate the effects on perceived pleasantness. No interaction effect Session* ASC-Daily, nor Velocity* ASC-Daily, nor Session*Velocity* ASC-Daily was found (all *p* > 0.281).

Similarly, an RM ANCOVA within-subjects [*Session*: 2 (Direct vs. Vicarious) and *Velocity*: 2 (AT; NAT)] was conducted, with ASC-12 in daily life (ASC-Daily) as a measure of cutaneous allodynia as the continuous covariate to evaluate the effects on perceived vividness. No interaction effect Session* ASC-Daily, nor Velocity* ASC-Daily, nor Session*Velocity* ASC-Daily was found (all *p* > 0.398).

##### Interplay Between Tactile Pleasant Affectivity and Pain Perception

A Pearson Correlation Matrix (N = 37) was used to evaluate the relationships among the perceived pleasant touch experience of direct and observed touch, pain perception (DP), vicarious pain (VP_INT; VP_N), and psychological variables (Table 1).

A significant positive correlation was found between the pleasantness of direct and observed AT (r = 0.776, *p* < 0.001), confirming consistency in hedonic touch perception across perceptual modalities. Moreover, a positive and significant correlation was found between pleasantness and vividness in vicarious AT (r = 0.363, *p* = 0.027), but not in direct AT (r = 0.1966, *p* = 0.244), highlighting how observed AT is perceived as more pleasant when experienced as more vivid. A positive correlation was found between the direct AT pleasantness and the vicarious AT vividness (r = 0.349, *p* = 0.034).

The significant negative correlations between the Social Touch Questionnaire (STQ) and the pleasantness of direct AT (r = −0.326, *p* = 0.049) and observed AT (r = −0.336, *p* = 0.042) suggested that a negative attitude toward social touch is related to a lower tactile pleasant affective experience. Significant positive correlations were found between BDI (r = 0.302, *p* = 0.049), STAI-Y2 (r = 0.536, *p* < 0.001) and STAI-Y1 (r = 0.620, *p* < 0.001) and STAI-Y2 with VP_N (r = 0.336, *p* < 0.042) (see Table 1).

The data suggest no mutual influence between pain and affective touch perception in direct and vicarious experience.

Pleasant AT interlaces the experience of being touched and watching someone else being touched. Additionally, the relationship between the vividness and pleasantness of AT is modality-dependent: they are correlated when the experience is vicarious, and there is an interaction between direct and vicarious conditions.

Furthermore, the hedonic experience of affective touch is related to variables of everyday social contact, attitude and emotional state.

However, no significant correlations emerged between experiencing pain on one’s skin and empathy for others’ pain.

These data and Table 1 report Pearson correlation coefficients computed across all measures. See the Supplementary Material for detailed data following a Holm adjustment for multiple comparisons.

## 3. Experiment 2: Migraine Population

### 3.1. Materials and Methods

The procedure of this second experiment is equal to that of the first experiment. See the previous experiment for all details.

#### 3.1.1. Participants

Thirty-seven participants (mean age: 49 years, SD = 15; age range: 18–71 years; 34 female) were enrolled at the Headache Centre, Neuroalgology Department of IRCCS Foundation “Carlo Besta” Neurological Institute of Milan, Italy. Patients (N = 37) included in the study presented a high frequency or history of chronic migraine with a disease duration of >24 months, without aura, and no medication overuse. The diagnosis was made according to the ICHD-3 criteria [47]. The mean frequency of symptomatic medication use was 9.0 ± 2.8 per month (median: 8; range: 6–15); this measure may be affected by the efficacy of the current treatment. A total of 73.1% of the participants used triptans only, 19.2% used NSAIDs only, and 7.7% used both triptans and NSAIDs for acute symptomatic treatment.

As preventive treatment, 47.8% of patients used anti-CGRP therapies, 21.7% amitriptyline, 17.4% botulinum toxin, 13.0% beta-blockers, and 4.3% topiramate; 4.3% were not receiving any preventive treatment. As some patients received combination preventive therapy, these percentages are not mutually exclusive.

None of the participants matched the following exclusion criteria: severe and documented medical or psychiatric conditions, epilepsy, sensory abnormalities such as multiple sclerosis and peripheral neuropathy, abnormal cutaneous perception or dermatological disease [31].

#### 3.1.2. Results

Thirty-seven participants completed all the questionnaires. Complete datasets were available for all 37 participants. 

Allodynia Symptom Checklist-12 (ASC-12):

ASC-12 assessed allodynia in daily life: (M = 2.35, SD = 3.58, range 0–12). Thirteen participants (35.1%) scored above the clinical threshold (≥3), reporting symptoms compatible with mild-to-moderate forms of allodynia. Additionally, ASC-12 was used to assess the presence of allodynia during the pain attack (M = 4.19, SD = 5.47, range 0–18). Fifteen participants (40.5%) scored above the clinical threshold (≥3). See the Supplementary Material for all detailed results (Text S2: EXPERIMENT 2: Data Analysis Supplemental Data).

##### Tactile Affectivity

First, the AT and NAT pleasantness and vividness mean intensity ratings were tested for normal distribution using the Shapiro–Wilk test. The test reported no significance for the variables. Bonferroni correction for the pairwise comparisons was used as required.

##### Pleasantness

A repeated-measures ANOVA (ANOVA RM) evaluated the effects of the touch condition [*Session*: 2 (Direct vs. Vicarious) and *Velocity*: 4 (AT = 3 and 6 cm/s; NAT = 18 and 30 cm/s)] on perceived pleasantness.

A main effect of *Session* was found [F(1, 36) = 6.75, *p* = 0.013, η^2^_p_ = 0.158]. Post hoc test comparisons indicated a significant difference [mean difference = 0.34, SE = 0.13, (t(36) = 2.60, *p* = 0.013)] (between perceived pleasantness in the Direct touch condition (M = 4.93, SE = 0.31) compared to the vicarious one (M = 4.59, SE = 0.27).

A main effect of *Velocity* also emerged [F(1.19, 42.95) = 48.14, *p* < 0.001, η^2^_p_ = 0.572].

The interaction for Session*Velocity was significant [F(2.02, 72.58) = 5.69, *p* = 0.005, η^2^_p_ = 0.137].

Bonferroni post hoc test comparisons between *velocities* revealed that slow AT velocities (3–6 cm/s) were perceived as significantly more pleasant than the faster NAT velocities (18–30 cm/s) (all *p* < 0.001).

There was no significant difference between AT 3 cm/s and 6 cm/s (*p* = 0.203), signifying that both CT-optimal velocities were perceived as the most pleasant and comparable.

Bonferroni post hoc analysis of the Session*Velocity interaction revealed a systematic decline in perceived pleasantness with increasing stimulation velocity, observed consistently across direct and vicarious modalities.

Direct AT 3 cm/s was rated as the most pleasant compared to direct and vicarious NAT 18 cm/s and to direct and vicarious NAT 30 cm/s (all *p* ≤ 0.002).

Likewise, direct AT 6 cm/s was rated as more pleasant compared to direct and vicarious NAT 18 cm/s and to NAT 30 (all *p* ≤ 0.001).

No significant mean differences were found among direct and vicarious AT experiences (all *p* = 1.00).

These data underscore the consistency of pleasantness perception at CT-optimal velocities of 3 cm/s and 6 cm/s in both direct and vicarious modes.

Differently, NAT 18 cm/s and NAT 30 cm/s velocities were affected by the different modalities of touch administration. NAT 18 cm/s was perceived as pleasant in the same way in the direct and in the vicarious session (*p* = 0.104), but NAT 30 cm/s was perceived as more pleasant in the direct mode than the vicarious one (*p* = 0.023). The results indicate that both stroking velocity and the administration modality significantly affect the perception of touch pleasantness. Affective touch (AT) velocities were consistently associated with higher pleasantness ratings across direct and vicarious conditions. In contrast, non-affective touch (NAT) velocities exhibited session-dependent effects: while pleasantness ratings for direct touch remained relatively stable, variations were observed in the vicarious modality (see Figure 6).

##### Pleasantness and Pain Perception

As in Experiment 1, three repeated-measures ANCOVAs (RM ANCOVAs) tested whether VP_INT, VP_N, or DP modulated AT/NAT vividness. Three repeated-measures ANCOVAs (RM ANCOVAs) evaluated the probability of the influence of direct or vicarious pain perception on the pleasantness of the overall AT (mean of 3 cm/s and 6 cm/s velocities) and NAT (mean of 18 cm/s and 30 cm/s velocities) tactile experience.

An RM ANCOVA within-subjects [*Session*: 2 (Direct vs. Vicarious) and *Velocity*: 2 (AT; NAT)] was conducted, adding VP Intensity (VP_INT), representing a measure of vicarious pain perception, as the continuous covariate to evaluate the effects on perceived pleasantness.

A main effect of *Velocity* was found [F(1, 35) = 21.57, *p* < 0.001, η^2^_p_ = 0.381]. Bonferroni post hoc test comparison revealed that AT (M = 5.77, SE = 0.32) was significantly perceived as more pleasant than NAT (M = 3.75, SE = 0.32) (*p* < 0.001).

A main effect of *Session* was found [F(1, 35) = 4.59, *p* = 0.039, η^2^_p_ = 0.116]. Bonferroni post hoc test comparison revealed that the direct modality (M = 4.93, SE = 0.31) was significantly perceived as more pleasant than vicarious (M = 4.59, SE = 0.28) (*p* = 0.015).

A significant interaction for Session*Velocity emerged [F(1, 35) = 4.97, *p* = 0.032, η^2^_p_ = 0.124].

The Bonferroni post hoc test showed that direct AT (M = 5.75, SE = 0.34) was rated more pleasant than direct NAT (M = 4.10, SE = 0.36) and vicarious NAT (M = 3.39, SE = 0.31). Likewise, vicarious AT (M = 5.79, SE = 0.33) was rated as more pleasant than both direct NAT and vicarious NAT.

These findings support the greater pleasantness of AT, which did not differ between direct and vicarious administration. Conversely, NAT pleasantness was higher in the direct than in the vicarious condition (See Figure 7).

An RM ANCOVA within-subjects [*Session*: 2 (Direct vs. Vicarious) and *Velocity*: 2 (AT; NAT)] was conducted, adding VP_N, the number of times participants perceived bodily pain while watching the VPQ videos, as the continuous covariate to evaluate the effects on perceived pleasantness.

A main effect of *Velocity* was found [F(1, 35) = 20.92, *p* < 0.001, η^2^_p_ = 0.374]. Bonferroni post hoc test comparison revealed that AT (M = 5.77, SE = 0.32) was significantly perceived as more pleasant than NAT (M = 3.75, SE = 0.32) (*p* < 0.001).

A main effect of *Session* was also found [F(1, 35) = 5.03, *p* = 0.031, η^2^_p_ = 0.126]. Bonferroni post hoc test comparison revealed that the direct modality (M = 4.93, SE = 0.31) was significantly perceived as more pleasant than vicarious (M = 4.59, SE = 0.28) (*p* = 0.014).

A significant interaction for Session*Velocity did not emerge [F(1, 35) = 1.43, *p* = 0.240, η^2^_p_ = 0.039].

The results of these two RM ANCOVAs indicate that individual sensitivity to vicarious pain perception did not affect the perception of tactile pleasantness.

Similarly, the direct pain perception (DP) variable was used as the covariate in the same RM ANCOVA model.to evaluate the effects on perceived pleasantness.

A main effect of *Velocity* emerged [F(1, 35) = 4.13, *p* = 0.005, η^2^_p_ = 0.105]. A Bonferroni post hoc test revealed a significant mean difference between AT (M = 5.77, SE = 0.32) and NAT (M = 3.75, SE = 0.32) (*p* < 0.001).

These results suggest that individual direct painful perception does not affect the tactile pleasantness experiences.

##### Vividness

A repeated-measures ANOVA (ANOVA RM) evaluated the effects of the touch condition [*Session*: 2 (Direct vs. Vicarious) and *Velocity*: 4 (AT = 3 and 6 cm/s; NAT = 18 and 30 cm/s)] on perceived vividness. Huynh–Feldt corrections were applied.

A main effect of *Session* emerged [F(1, 36) = 22.79, *p* < 0.001, η^2^_p_ = 0.388].

A main effect of *Velocity* was not found [F(1.16, 41.86) = 3.01, *p* = 0.085, η^2^_p_ = 0.077].

The interaction for Session*Velocity was significant [F(2.44, 88.02) = 4.54, *p* = 0.009, η^2^_p_ = 0.112].

Bonferroni post hoc test comparisons between *sessions* revealed that direct touch (M = 6.08, SE = 0.38) was significantly more vivid than vicarious (M = 4.35, SE = 0.34) (*p* < 0.001).

A Bonferroni post hoc test for the interaction Session*Velocity suggested that the perceived vividness is homogeneous within the administration modality: no significant differences in vividness among all the velocities within the observed or direct sessions were found (all *p* > 1.000). However, significant differences emerged between the direct and corresponding vicarious modality at AT 3 cm/s (*p* = 0.020), AT 6 cm/s (*p* = 0.007), NAT 18 cm/s (*p* < 0.001), and NAT 30 cm/s (*p* < 0.001).

Overall, vividness was primarily determined by stimulation modality, with direct touch rated as more vivid than observed touch (see Figure 8).

##### Vividness and Pain Perception

Three repeated-measures ANCOVAs (RM ANCOVAs) assessed the effect of direct or vicarious pain perception on the vividness of AT (mean of 3 cm/s and 6 cm/s velocities) and NAT (mean of 18 cm/s and 30 cm/s velocities) tactile experiences.

An RM ANCOVA within-subjects [*Session*: 2 (Direct vs. Vicarious) and *Velocity*: 2 (AT; NAT)] was conducted, adding VP Intensity (VP_INT), the vicarious pain perception, as the continuous covariate to evaluate the effects on perceived vividness.

A main effect of *Session* emerged [F(1, 35) = 14.128, *p* < 0.001, η^2^_p_ = 0.288]. Bonferroni post hoc test comparison shows that direct touch (M = 6.08, SE = 0.38) is more vivid than vicarious (M = 4.35, SE = 0.35) [mean difference = 1.74, SE = 0.37, (t(35) = 4.71, *p* < 0.001)].

A main effect of *Velocity* was not found [F(1, 35) = 0.167, *p* = 0.0685, η^2^_p_ = 0.005].

An RM ANCOVA within-subjects [*Session*: 2 (Direct vs. Vicarious) and *Velocity*: 2 (AT; NAT)] was conducted, adding VP_N, which represents the number of times participants perceived bodily pain in vicarious pain experiences.

A main effect of *Session* emerged [F(1, 35) = 13.97, *p* < 0.001, η^2^_p_ = 0.285]. Bonferroni post hoc test comparison confirmed that direct touch is more vivid than vicarious [mean difference 1.74, SE = 0.37, (t(35) = 4.73, *p* < 0.001)].

Another RM ANCOVA within-subjects analysis [*Session*: 2 (Direct vs. Vicarious) and *Velocity*: 2 (AT; NAT)] was conducted, adding direct pain perception (DP) as a continuous covariate to evaluate the effects on perceived vividness.

A main effect of *Session* was found [F(1, 35) = 9.46, *p* = 0.004, η^2^_p_ = 0.213]. Bonferroni post hoc test comparison confirmed that direct touch is more vivid than vicarious [mean difference = 1.74, SE = 0.37, (t(35) = 4.83, *p* < 0.001)].

An interaction effect for Velocity* *Session* emerged [F(1, 35) = 5.83, *p* = 0.021, η^2^_p_ = 0.059].

The Bonferroni post hoc test comparison confirmed that direct AT (M = 6.26, SE = 0.38) is more vivid than vicarious AT (M = 4.82, SE = 0.36) (*p* = 0.002); direct NAT (M = 5.91, SE = 0.46) is more vivid than vicarious NAT (M = 3.88, SE = 0.39) (*p* < 0.001); direct AT is more vivid than vicarious NAT (*p* < 0.001) (see Figure 9).

##### Pleasantness and Cutaneous Allodynia

We also investigate the role of perceived cutaneous allodynia in the pleasantness and vividness experienced with touch.

An RM ANCOVA within-subjects [*Session*: 2 (Direct vs. Vicarious) and *Velocity*: 2 (AT; NAT)] was conducted, adding ASC-12 in daily life (ASC-Daily) as a measure of cutaneous allodynia as the continuous covariate to evaluate the effects on perceived pleasantness. There was an interaction effect of Session* ASC-Daily in daily life [F(1, 35) = 10.30, *p* = 0.003, η^2^_p_ = 0.227]. No other interaction effect of Velocity* ASC-Daily, nor Session*Velocity* ASC-Daily, was found (all *p* > 0.539).

An RM ANCOVA within-subjects [*Session*: 2 (Direct vs. Vicarious) and *Velocity*: 2 (AT; NAT)] was conducted, adding ASC-12 during the pain attacks (ASC-PA) as a measure of cutaneous allodynia as the continuous covariate to evaluate the effects on perceived pleasantness. The analysis results replicate the previous ANCOVA’s results. There was an interaction effect for Session* ASC-PA [F(1, 35) = 5.9, *p* = 0.028, η^2^_p_ = 0.131]. No other interaction effect of Velocity* ASC-PA, nor Session*Velocity* ASC-PA, was found (all *p* > 0.614).

##### Interplay Between Tactile Pleasant Affectivity and Pain Perception

A Pearson Correlation Matrix (N = 37) was used to explore the relationships among the perceived hedonic experience of direct and observed touch, direct pain perception, vicarious pain (VP_N and VP_INT), and psychological variables (see Table 2). The Holm correction for multiple correlations was applied.

A significant positive correlation was found between the pleasantness of direct and vicarious AT (r = 0.865, *p* < 0.001) and between the vividness of direct and vicarious AT (r = 0.538, *p* < 0.001), confirming the consistency in affective touch perception across perceptual modalities. Positive significant correlations were found between the direct AT pleasantness and direct vividness (r = 0.761, *p* < 0.001), and direct AT pleasantness and vicarious vividness (r = 0.754, *p* < 0.001).

Direct pain perception was found to be negatively correlated with state (STAI-Y1) (r = −0.346, p = 0.036) and trait anxiety (STAI-Y2) (r = −0.411, p = 0.011).

STQ score was positively correlated with the presence of allodynia during the pain attacks (ASC-PA) (r = 0.468, *p* = 0.003) and BDI (r = 0.426, *p* = 0.008), as well as STAI-Y1 (r = 0.497, *p* = 0.002) and STAI-Y2 (r = 0.721, *p* < 0.001).

Significant positive correlations were found between the BDI and anxiety indexes STAI-Y2 (r = 0.767, *p* < 0.001) and STAI-Y1 (r = 0.571, *p* < 0.001) (see Table 2).

The correlation matrix shows the relationship between direct and vicarious AT pleasantness. Additionally, the vividness and pleasantness of AT are not modality-dependent. The pleasant AT experience of being touched and of watching someone else being touched is positively related to the perception of vividness of those experiences.

The allodynia symptoms seem unrelated to either the AT experience or the pain in this experimental context. However, allodynia during pain attacks (ASC-PA) is positively linked to the avoidance of social touch in daily life (STQ) that, in turn, is positively related to depression (BDI) and anxiety (STAI-Y1, STAI-Y2).

The positive correlations between mood variables (BDI, STAI-Y1, and STAI-Y2) confirm their interplay in chronic pain conditions.

The data and Table 2 refer to Pearson correlation coefficients computed across all measures. Detailed data following a Holm adjustment for multiple comparisons are provided in the Supplementary Materials.

### 3.2. Pain and Pleasantness Comparison Between the Two Populations

The previous analyses in healthy populations and migraine sufferers suggested similar patterns in tactile experience pleasantness, but some differences in pain perception.

Thus, further analysis was deemed necessary to compare the two populations and add more detail to the discussion.

A preliminary *t*-test revealed a significant age difference between groups (*p* < 0.001). A χ^2^-Test did not show a significant sex difference between groups (*p* = 0.102).

A repeated-measures ANOVA (ANOVA RM) evaluated the effects of the touch condition [*Session*: 2 (Direct vs. Vicarious) and *Velocity*: 2 (AT; NAT)] on perceived pleasantness in two groups: a healthy population (H) and a population of migraineurs (CM).

A main effect of *Session* emerged [F(1, 72) = 8.26, *p* = 0.005, η^2^_p_ = 0.103].

A main effect of *Velocity* was found [F(1, 72) = 119.43, *p* < 0.001, η^2^_p_ = 0.624].

The interaction for Session*Velocity was significant [F(1, 72) = 26.30, *p* < 0.001, η^2^_p_ = 0.268].

No other interaction effects were found (all *p* > 0.165).

These findings support the evidence that CM patients did not diverge from the healthy population, as their chronic pain does not seem to affect their perception of pleasant touch.

Also, the pain perception scores were compared in detail between the two populations.

Normal distributions of each variable were tested via the Kolmogorov–Smirnov test. Therefore, between-groups differences in ASC-Daily and VP_INT were investigated using the non-parametric independent samples Mann–Whitney U test. In contrast, T, VP_N, and DP were examined via the independent samples *t*-test.

The analysis revealed a significant mean difference in pain threshold (T) between H (M = 2.93, SD = 2.48) and CM (M = 7.92, SD = 3.94): t = *p* < 0.001, d = −1.336.

Also, there was a significant difference in VP_N between H (M = 7.11, SD = 4.36) and CM (M = 3.89, SD = 3.80): t = *p* = 0.001, d = 0.787.

VP_INT difference was at the limit of significance, u = *p* = 0.055, r = 0.2564, for H (M = 3.70, SD = 2.65) and CM (M = 2.59, SD = 2.99).

No significant difference emerged in ASC-Daily and DP between the groups (*p* > 0.337).

These findings revealed that CM patients have a higher pain threshold and are less sensitive to others’ pain than the healthy population.

### 3.3. General Discussion

The present research project assessed the direct and vicarious somatosensory experiences of affective touch and pain and explored their relationship in healthy and migraineur (CM) populations. Both groups’ results confirmed our first predictions, showing that AT, at 3 cm/s and 6 cm/s, is perceived as more pleasant than NAT, supporting the involvement of C-tactile fibres in mediating the hedonic dimension of slow affective touch, as in the current literature [9,21,25,43,48]. AT also produced a higher pleasantness rating than NAT in both direct and observed administration. Also, pleasantness perception related to stroking velocity followed the usual U-shaped pattern [25]. For the first time, we found that direct and observed AT can induce similar hedonic responses. In healthy and CM populations, the similarity of hedonic ratings between direct and observed AT suggests that vicarious touch can evoke affective resonance akin to actual physical contact, likely engaging shared neurocognitive mechanisms such as sensory simulation and social-affective mirroring [21], and the central role of observation in sensory simulation and empathic tactile processes [22]. Furthermore, vicarious AT was found to be more pleasant than direct NAT, suggesting that the intrinsic affective quality of the stimulus can persist beyond the somatosensory immediacy of direct perception. This finding may be relevant to support the use of AT interventions, suggested as a tool for improving affective conditions as well as pain perception [49,50], even in people who dislike being touched, those with cutaneous allodynia or those affected by pain conditions accompanied by reduced affective touch mechanisms [51].

In CM, despite a prevalence of cutaneous allodynia, the pleasantness of affective touch is confirmed to be relatively preserved [28], particularly in the direct modality, even though allodynia modulated the subjective experience of touch. This preservation could indicate a compensatory or dissociative mechanism, where affective touch continues to function as a potential source of positive sensory input despite altered nociceptive processing. The perception of vividness across groups was more dependent on the modality (direct vs. observed) than on velocity (AT vs. NAT), underscoring the role of direct somatosensory engagement in shaping the intensity of tactile experiences. Individual differences in direct pain sensitivity significantly modulated this vividness in the healthy population, consistent with interoceptive models of somatic amplification [1,52], which state that heightened somatic responsiveness can amplify the intensity of even non-noxious stimuli. In contrast, empathy for others’ pain modulated the vividness of touch in CM; nevertheless, there were no significant correlations between vicarious pain and AT vividness. Intriguingly, individuals in both clinical and non-clinical populations experienced allodynia. This finding highlights the importance of considering allodynia within the general population as a potential variable influencing pain and affective touch perception; however, this study found no evidence to support this. However, the presence of allodynia during pain attacks in CM was positively related to social touch avoidance, suggesting an underlying defensive reaction against cutaneous hypersensitivity via avoidance of interpersonal touch [53]. Social touch avoidance had a different impact on the two populations. In the healthy group, it was related to affective touch perception. In CM patients, it was more closely associated with mood traits, suggesting a complex interplay between individual behaviour and interpersonal context. Direct comparison between healthy (H) and migraineur (CM) populations regarding pain and AT perception allowed for further speculation on whether these processes may be atypical in chronic pain conditions. AT experiences were similar in both populations; chronic pain did not seem to affect their perception. Moreover, affective touch findings revealed a significant correlation between direct and vicarious experiences, with no notable differences in perceived pleasantness across these conditions, consistent with the insula’s purported key role in the mechanisms underlying vicarious perception of pleasant touch [29]. In contrast to AT perception, no significant relationship was found between responses to others’ pain and the subjective experience of first-hand pain. This suggests that the cognitive and affective mechanisms underlying pain empathy may be distinct from those engaged during the direct experience of pain. It can explain the lack of a clear relation between direct and vicarious pain in both populations [54]. Nevertheless, the two groups’ pain thresholds (T) and vicarious pain differed significantly. CM showed a higher T, even if the response to direct painful stimuli did not diverge substantially from that of healthy people. Interestingly, CM patients appeared to be significantly less empathetic to others’ pain. These findings suggest that while affective touch perception remains largely preserved in migraine, differences emerge in social and empathic dimensions related to pain. It must be noted that, while vicarious AT stimuli were video representations of a real hand stroking, direct pain experiences could not replicate the scenes shown in the vicarious pain questionnaire, which may have influenced the results. The observed associations between allodynia, social touch avoidance, mood traits, and reduced empathy for others’ pain indicate that migraine may influence the interpersonal and affective aspects of pain processing more than the hedonic perception of affective touch itself. Chronic pain sufferers may exhibit avoidance behaviours, leading to reduced social cognition, including empathy, which may either precede the onset of chronic pain or develop as a consequence, ultimately playing a role in the persistence of pain and hindering recovery [54,55,56]. This model may be supported by the evidence in this study of the positive correlation between avoidance of social touch in daily life and allodynia during pain attacks, and with depression and anxiety in the CM population.

Overall, although no physiological measures (e.g., skin conductance response) were evaluated to complement subjective reports and the data were collected in an experimental setting, the pleasantness of affective touch was preserved despite altered somatosensory processing. These behavioural results further expand knowledge of the potential clinical applications of affective touch as a therapeutic intervention for chronic pain [24].

They also highlight the importance of personalised approaches that consider both sensory and psychological dimensions of touch experiences.

To summarise, this study advances the understanding of affective touch by demonstrating that:

- Affective touch maintains its hedonic quality across both healthy and migraine populations.
- Pain and affective touch perception are not significantly related under the specific conditions of this study and in the participants evaluated.
- The modulation of touch vividness is shared across groups and is not influenced by the modality and pain sensitivity, with a limited influence of vicarious pain in the healthy population.
- Psychological traits may be influential in both populations (see Table 3).

One promising avenue is the use of immersive virtual reality to simulate affective touch in chronic pain populations. Visual simulations of gentle touch, embodied in a virtual body, can elicit pleasant responses and activate interoceptive and affective processing pathways, even without real tactile stimulation [29,57]. These findings highlight the influence of visually mediated touch on affective and somatosensory processing and its potential therapeutic relevance in clinical populations. Moreover, this could be further enhanced by accounting for relational or contextual variables, such as familiarity, trust, or social context, which are known to influence affective responses to touch [58,59].

Additionally, there was no evidence of a significant relationship between pain and affective touch under the specific conditions of this study and in the participants evaluated. We can speculate that they do not influence each other; they appear to be processed independently, even though they share some neurophysiological mechanisms. Individual differences in pain perception and empathic responsiveness do not substantially influence affective evaluation; this is likely due to their different natures: while pain is more alerting, touch is more focused on interpersonal relationships.

This research primarily aimed to evaluate the influence of pain on affective touch perception. It also contributed to the enrichment of knowledge about the relationships among pain, affective touch, allodynia, and social behaviour measures. There are some limitations to note, and additional explanations to add to improve the data’s conclusions. The self-report screening criteria in the healthy group may have allowed the inclusion of participants with subclinical conditions and accounted for the presence of allodynia, even in the absence of documented sensitisation or chronic pain. Cutaneous allodynia arises from a constellation of alterations in both the peripheral and central nervous systems and may be present in healthy individuals, more likely modulated by cognitive and emotional factors. Psychological stress and emotional states are also known to influence pain processing via descending pathways, amplifying sensory input and possibly unmasking latent allodynia [60]. So, the proportion of control participants who reported symptoms consistent with cutaneous allodynia, according to the ASC-12 criteria, does not directly imply the presence of somatosensory sensitisation. Also, the ASC-12 is a self-report measure; it identifies symptoms consistent with allodynia but cannot by itself establish the presence of a specific sensitisation mechanism. Further investigation of this aspect would require more specific sensory and/or physiological measures. Nevertheless, these events may have affected the overall data, particularly by amplifying the direct pain experience. Further studies may be needed to deepen the understanding of allodynia in the healthy population and the mechanisms underlying pain and affective touch perception, prompting a new research project and further speculation.

The age difference between groups must be taken into consideration. Ageing may differentially affect the various components of tactile perception: while sensory-discriminative aspects tend to decline with age, the affective component of touch may be relatively enhanced, especially above 60 years [61,62]. Nevertheless, no significant effect of age on pleasantness ratings was found in the 15–55-year age range [63]. The link between age and pain perception is controversial. With advancing age, pain sensitivity to low-intensity stimuli decreases, as reflected by an increased pain threshold, whereas pain tolerance remains largely unchanged [64]. Older age was associated with a higher pressure pain threshold, whereas age-related effects on other pain measures were less consistent across age groups [65,66]. Regarding the discrepancy in the number of female participants between the two groups, some explanation is needed. Although there is no significant sex difference between the groups, the predominance of female participants warrants exploration. Migraine is a condition which shows a higher incidence of disease in women: this research project tried to reproduce the gender-related differences [67]. Female gender prevalence must be considered as a potential influencing factor on the results. However, evidence that affective touch perception can be sex-related remains controversial. A slightly greater touch pleasantness in women than in men was found, suggesting a modest difference in the affective evaluation of touch rather than in general tactile sensitivity [68]. Nevertheless, no significant sex differences in sensory pleasantness were found when touch was experimentally controlled, while differences emerged primarily in social and contextual aspects of affective touch [69]. These findings suggest that sex-related differences may reflect the social and affective interpretation of touch rather than fundamental differences in sensory processing. Regarding sex differences in pain perception, sex differences in pain perception appear to reflect both biological and psychosocial factors. A recent meta-analysis found higher pain thresholds and tolerance, but lower perceived pain intensity, in males than in females [70]. Importantly, gender-role expectations explained additional variance in pain threshold, tolerance, and unpleasantness, with their contribution being particularly pronounced for the affective dimension of pain [71]. So, these findings highlight the need to consider both sex-related and gender-related factors when interpreting individual differences in pain responses.

Also, a larger sample size in the pain and pleasantness comparisons between the two populations could have revealed more robust results. The extensive use of exploratory analyses, intended to clarify the working hypotheses, may have increased the risk of statistical errors, even with some corrections in place.

In conclusion, this research study highlights that the body is not a passive receptor of stimuli, but rather a site of meaning-making in interactions with both the internal and external worlds, where vulnerability, relational predispositions, and perceptual processing capacities converge.

## Figures and Tables

**Figure 1:**
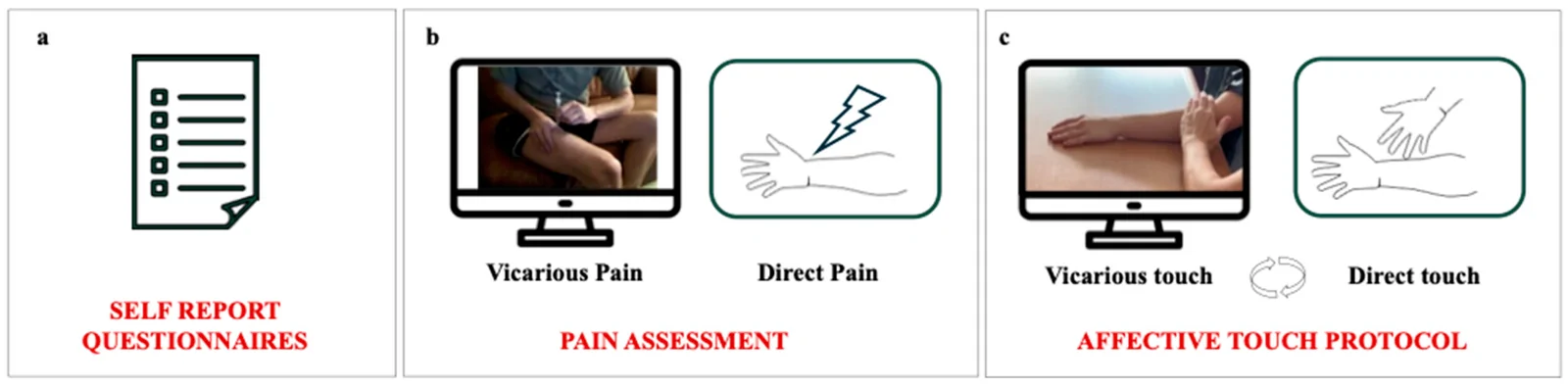
Schematic representation of the study procedure: (**a**) 1° phase: psychometric assessment; (**b**) 2° phase: direct and vicarious pain assessment; (**c**) 3° phase: direct and vicarious affective touch sessions in randomised order.

**Figure 2:**
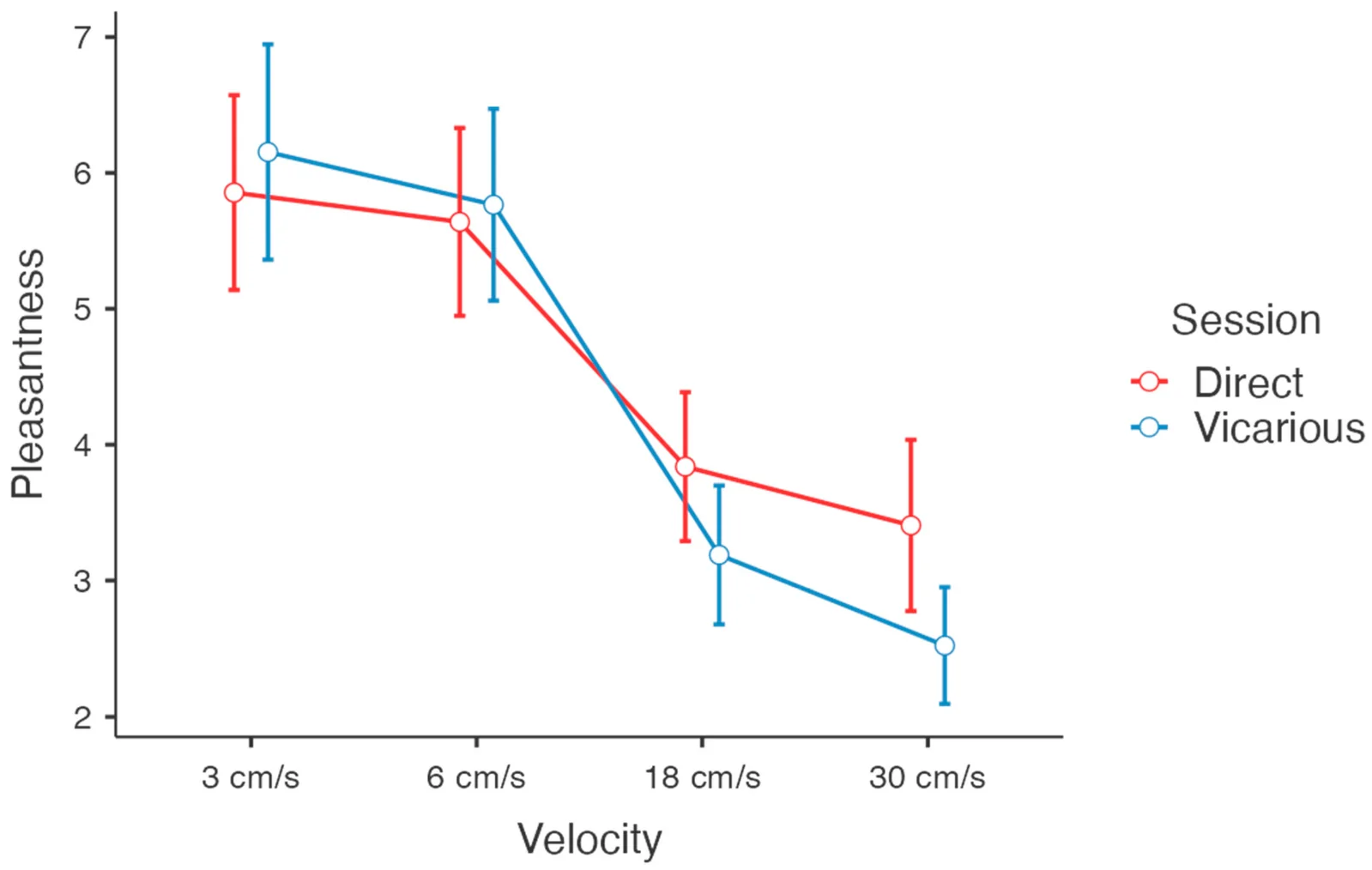
Graphic representation of perceived pleasantness of touch as a function of session type (Direct vs. Vicarious) and stroking velocity (AT: 3 cm/s and 6 cm/s; NAT: 18 cm/s and 30 cm/s). Slow AT velocities (3–6 cm/s) were significantly more pleasant than the faster NAT velocities (18–30 cm/s) (all *p* < 0.001). Direct and vicarious AT 3 cm/s were perceived as more pleasant compared to direct and vicarious NAT 18 cm/s (*p* < 0.001) and NAT 30 cm/s (*p* < 0.001). Direct and vicarious AT 6 cm/s were rated more pleasant than direct and vicarious NAT 18 cm/s (*p* < 0.001) and NAT 30 cm/s (*p* < 0.001). While perceived pleasantness between AT 3 cm/s and AT 6 cm/s in direct and vicarious sessions was similar, NAT 18 cm/s (*p* = 0.003) and NAT 30 cm/s velocities were perceived more pleasant in the direct session than in the vicarious one (*p* = 0.01).

**Figure 3:**
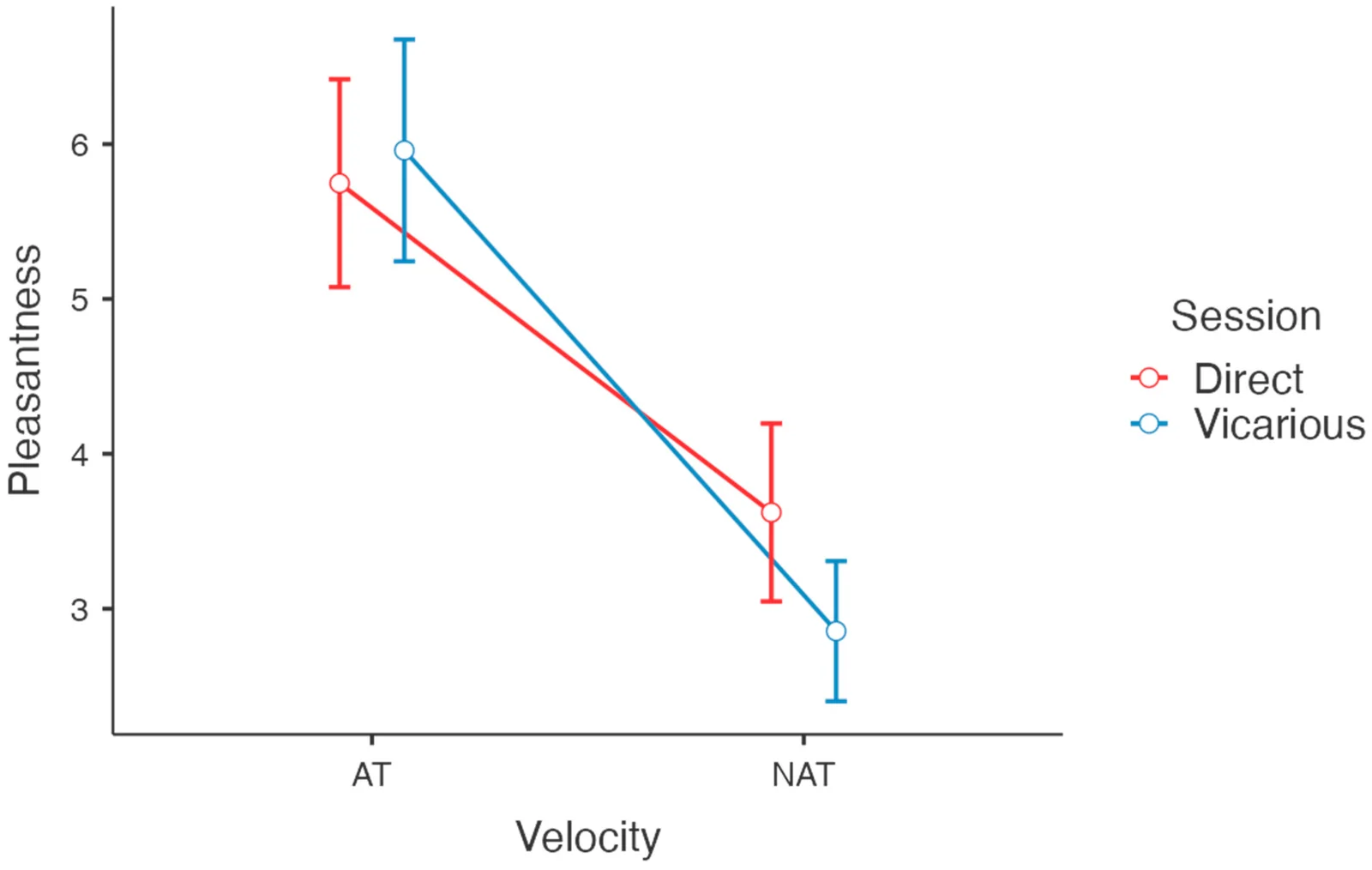
Graphic representation of perceived pleasantness of affective touch (AT) and non-affective touch (NAT) in function of session type (Direct vs. Vicarious). AT was significantly perceived as more pleasant than NAT in both direct and vicarious modalities (*p* < 0.001).

**Figure 4:**
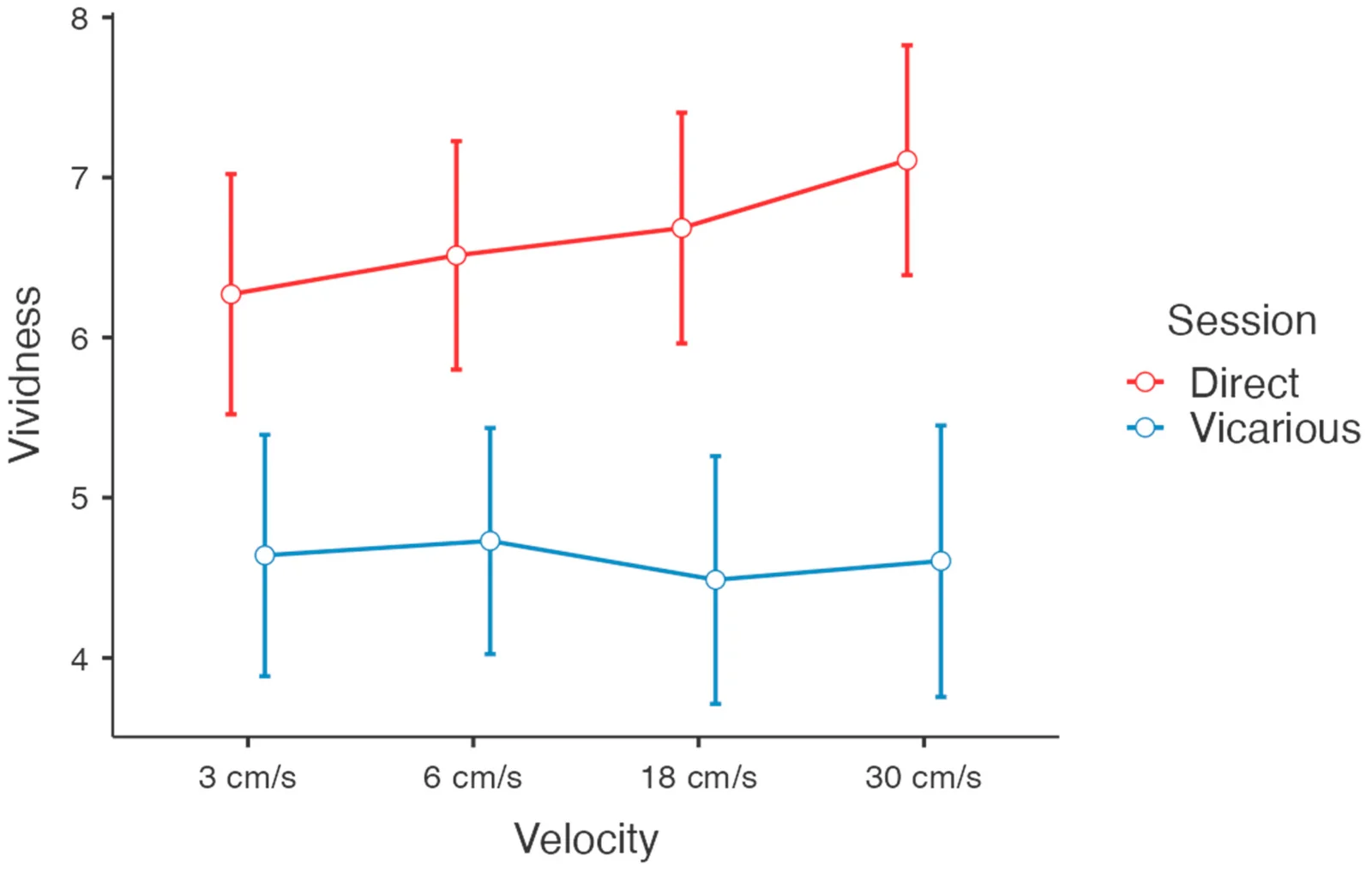
Graphic representation of perceived vividness of touch as a function of session type (Direct vs. Vicarious) and stroking velocity (AT: 3 cm/s and 6 cm/s; NAT: 18 cm/s and 30 cm/s).

**Figure 5:**
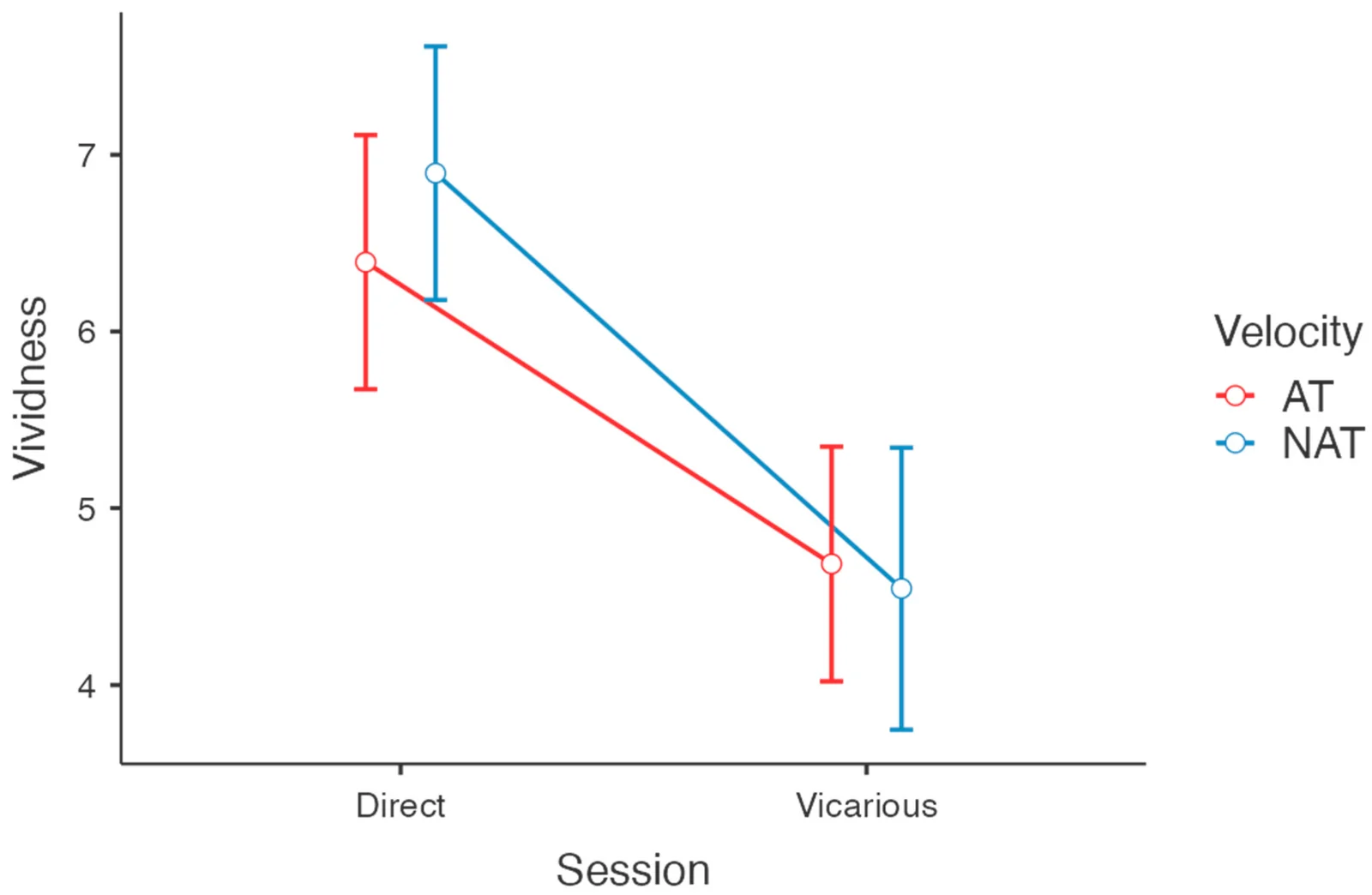
Graphic representation of perceived vividness of affective touch (AT) and non-affective touch (NAT) in function of session type (Direct vs. Vicarious). Direct touch was significantly perceived as more vivid than the vicarious one (*p* < 0.001).

**Figure 6:**
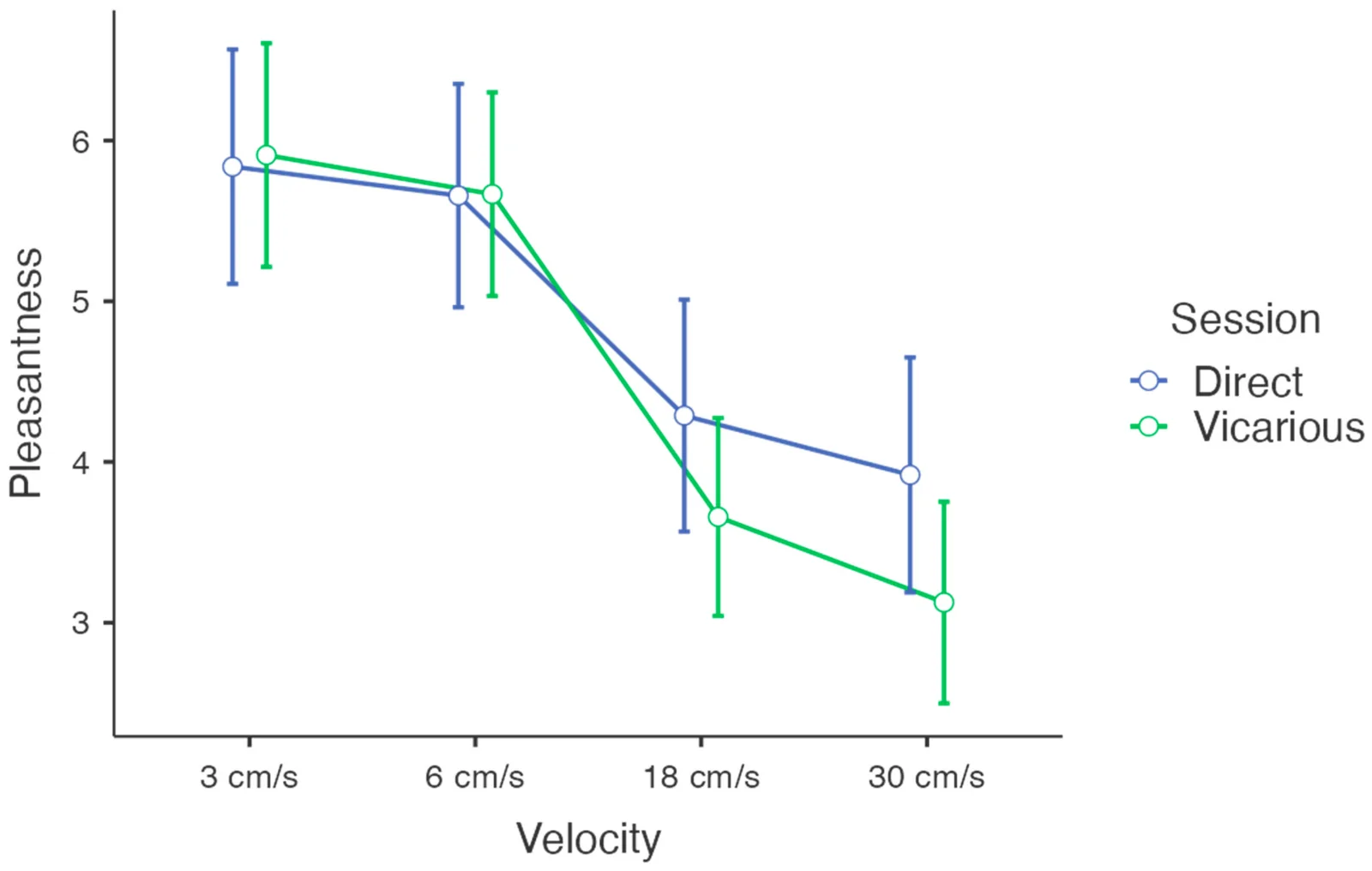
Graphic representation of perceived pleasantness of touch as a function of session type (Direct vs. Vicarious) and stroking velocity (AT: 3 cm/s and 6 cm/s; NAT: 18 cm/s and 30 cm/s).

**Figure 7:**
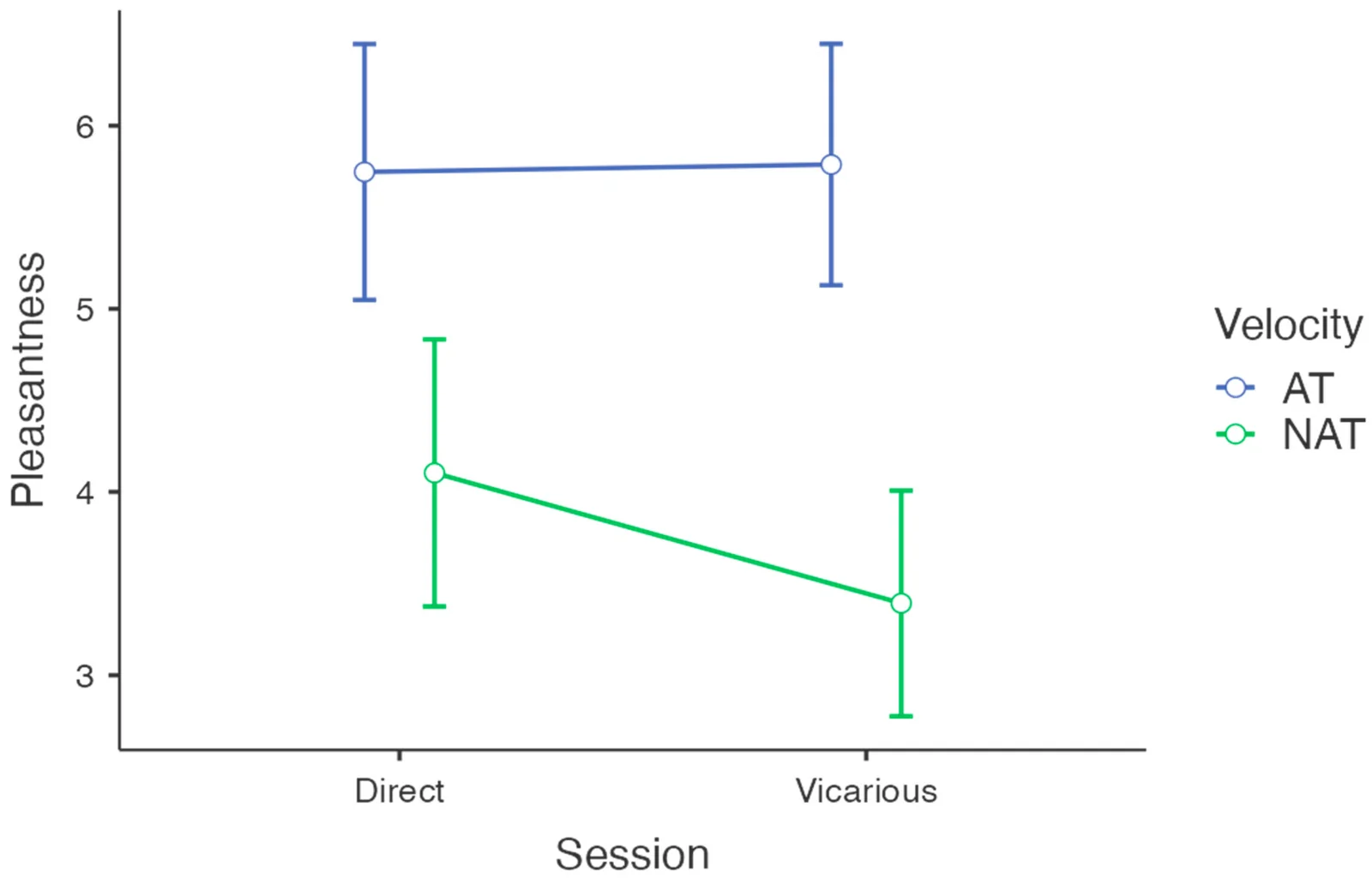
Graphic representation of perceived pleasantness of affective touch (AT) and non-affective touch (NAT) in function of session type (Direct vs. Vicarious).

**Figure 8:**
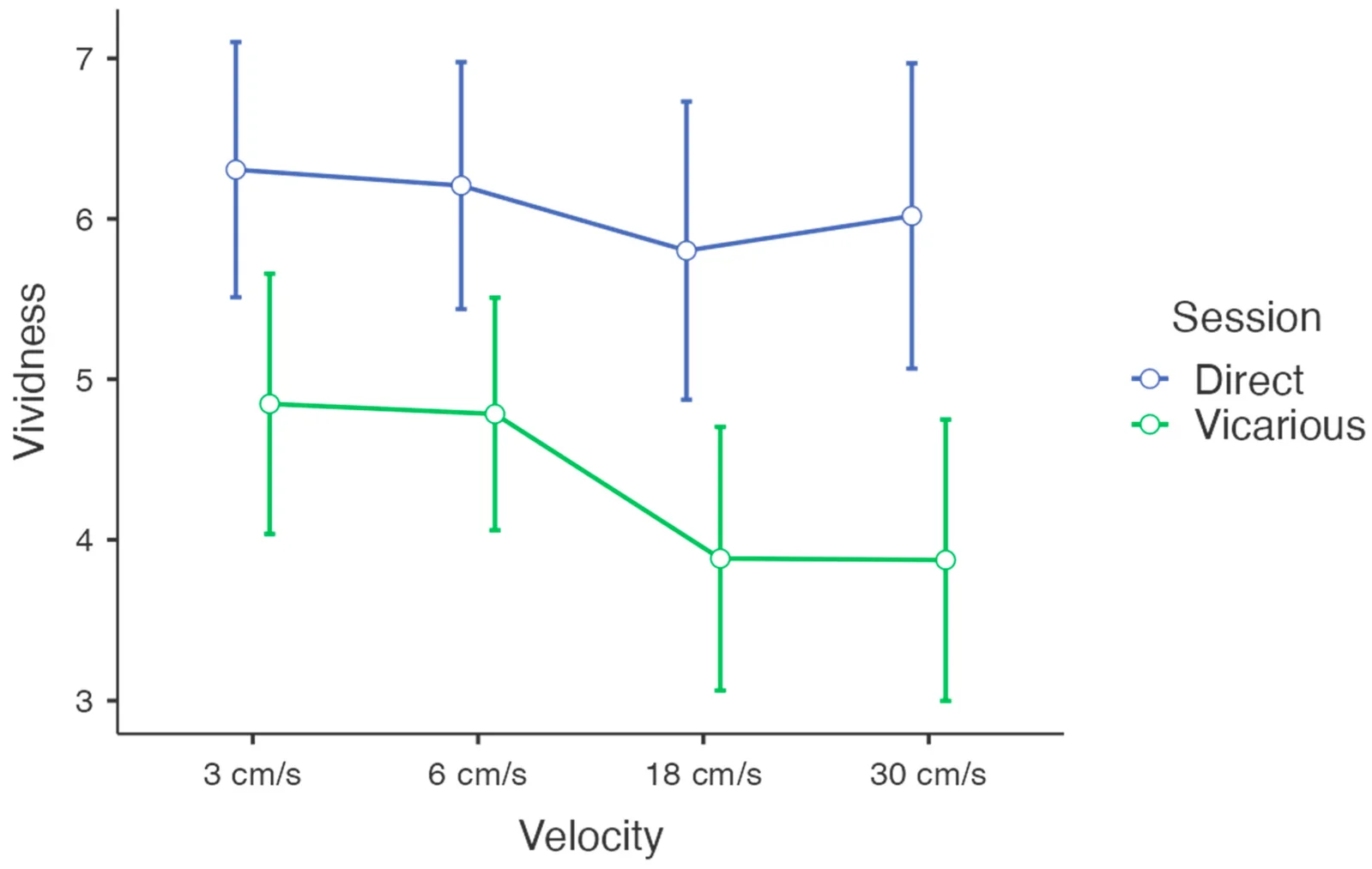
Graphic representation of perceived vividness of touch as a function of session type (Direct vs. Vicarious) and stroking velocity (AT: 3 cm/s and 6 cm/s; NAT: 18 cm/s and 30 cm/s).

**Figure 9:**
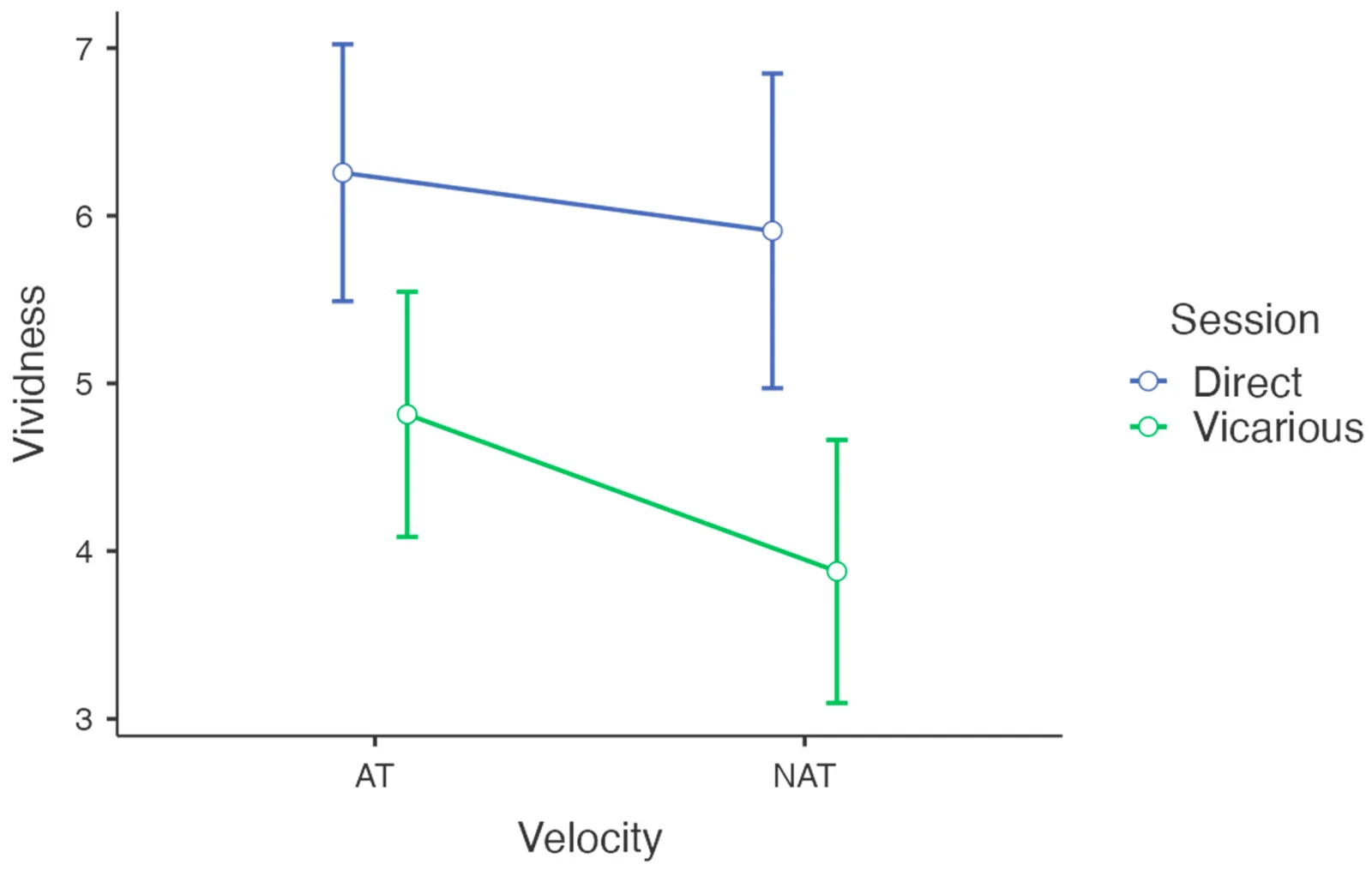
Graphic representation of perceived vividness of affective touch (AT) and non-affective touch (NAT) in function of session type (Direct vs. Vicarious).

**Table 1:** Correlation matrix of the main study variables. The variables include: direct pain; vicarious pain intensity (VP_INT); times of perceived vicarious pain (VP_N); direct AT pleasantness (AT_DPLS); vicarious AT pleasantness (AT_VPLS); direct AT vividness (AT_DVIV); vicarious vividness (AT_VVIV); allodynia symptoms in daily life (ASC-Daily); social touch avoidance (STQ); depression (BDI); state anxiety (STAI-Y1); trait anxiety (STAI-Y2). Statistically significant correlations are marked with asterisks (* *p* < 0.05, *** *p* < 0.001).

	Direct Pain	VP_N	VP_ INT	AT_DPLS	AT_DVIV	ASC- Daily	STQ	AT_VPLS	AT_VVIV	BDI	STAY Y1	STAY Y2
Direct Pain	—											
VP_N	0.286	—	0.669 ***	0.157	−0.103	0.014	−0.035	0.074	0.220	0.082	0.214	0.336 *
VP_INT	0.172	0.669 ***	—									
AT_DPLS	0.173	0.157	0.174	—								
AT_DVIV	−0.078	−0.103	0.144	0.196	—							
ASC-Daily	−0.05	0.014	0.002	0.068	−0.096	—						
STQ	0.320	−0.035	0.183	−0.326 *	0.099	0.061	—					
AT_VPLS	0.131	0.074	0.131	0.776 ***	0.072	0.166	−0.336 *	—				
AT_VVIV	0.264	0.220	0.374 *	0.349 *	0.379 *	−0.037	−0.023	0.363 *	—			
BDI	−0.039	0.082	0.170	−0.207	0.010	0.315	0.321	−0.275	−0.027	—		
STAY Y1	0.105	0.214	0.059	−0.15	0.034	0.022	0.287	−0.244	0.007	0.620 ***	—	
STAY Y2	0.295	0.336 *	0.281	0.057	0.236	0.243	0.206	0.075	0.239	0.536 ***	0.387 *	—

**Table 2:** Correlation matrix of the main study variables. The variables include: direct Pain; vicarious pain intensity (VP_INT); times of perceived vicarious pain (VP_N); direct AT pleasantness (AT_DPLS); vicarious AT pleasantness (AT_VPLS); direct AT vividness (AT_DVIV); vicarious vividness (AT_VVIV); allodynia symptoms in daily life (ASC-Daily); allodynia symptoms in pain attacks (ASC-PA); social touch avoidance (STQ); depression (BDI); state anxiety (STAI-Y1); trait anxiety (STAI-Y2). Statistically significant correlations are marked with asterisks (* *p* < 0.05, ** *p* < 0.01, *** *p* < 0.001).

	VP_INT	Direct Pain	AT_DPLS	AT_VPLS	AT_DPVIV	AT_VVIV	ASC-Daily	ASC-PA	STQ	BDI	STAY-Y1	STAY-Y2	VP_N
VP_INT	—												
Direct Pain	−0.285	—											
AT_DPLS	0.121	0.005	—										
AT_VPLS	0.140	0.050	0.865 ***	—									
AT_DPVIV	0.026	−0.201	0.761 ***	0.600 ***	—								
AT_VVIV	0.121	0.093	0.754 ***	0.848 ***	0.538 ***	—							
ASC-Daily	−0.053	0.028	0.288	0.074	0.200	−0.028	—						
ASC-PA	0.066	−0.009	0.132	−0.035	0.190	−0.163	0.844 ***	—					
STQ	0.267	−0.206	−0.300	−0.299	−0.184	−0.267	0.322	0.468 **	—				
BDI	0.232	−0.277	0.135	0.091	0.273	0.113	0.273	0.270	0.426 **	—			
STAY-Y1	0.034	−0.346 *	−0.123	−0.206	0.011	−0.048	0.232	0.168	0.497 **	0.571 ***	—		
STAY-Y2	0.265	−0.411 *	−0.162	−0.217	−0.063	−0.187	0.274	0.305	0.721 ***	0.767 ***	0.779 ***	—	
VP_N	0.723 ***	−0.156	−0.127	−0.016	−0.185	−0.058	−0.144	0.087	0.321	0.153	0.087	0.268	—

**Table 3:** Comparison of key findings of main variables in the healthy (H) and migraine (CM) group.

ASPECT	HEALTHY POPULATION	CM POPULATION
AT PLEASANTNESS (AT PLS)	Higher pleasantness at AT velocities. Similarity between direct and observed AT.	Higher pleasantness at AT velocities. Similarity between direct and observed AT.
AT VIVIDNESS	Direct touch is more vivid than observed.	Direct touch is more vivid than observed.
PAIN − AT PLS	No influence of DP or VP on AT pleasantness.	No influence of DP or VP on AT pleasantness
DIRECT PAIN (DP)	No Influence on pleasantness or vividness.	Did not influence pleasantness.
VICARIOUS PAIN (VP)	No influence on pleasantness Influence on vividness by velocity	No influence on touch pleasantness or vividness
ALLODYNIA (ASC−Daily; ASC−PA)	27% of participants present daily allodynia. No influence on AT perception.	ASC−Daily in 35% of participants. ASC−PA in 40% of participants: light influence on pleasantness/vividness perception depending on the touch modality
PSYCHOLOGICAL VARIABLES	Correlations: (−) social touch avoidance −AT PLS (+) anxiety−VP_N	Correlations: (−) Anxiety−DP (+) social touch avoidance−ASC−PA (+) social touch avoidance with BDI and Anxiety

## Data Availability

The original contributions presented in this study are included in the article/Supplementary Materials. Further inquiries can be directed to the corresponding author(s).

## Supplementary Materials

The following supporting information can be downloaded at: https://www.mdpi.com/article/10.3390/brainsci16101009/s1, Text S1: EXPERIMENT 1: Data Analysis Supplemental Data; Text S2: EXPERIMENT 2: Data Analysis Supplemental Data.
