# Supplementary material for: Pain Sensitivity and Affective Touch Perception: Evidence from Direct and Vicarious Experiences in Healthy Individuals and Patients with Migraine
Source: Brain Sci. 2026 Sep 23;16(10):1009. doi: 10.3390/brainsci16101009 (PMC13702440; doi:10.3390/brainsci16101009)
Supplement: Supplementary file 1 [file brainsci-16-01009-s001.zip › brainsci-4438979-supplementary.pdf]

Supplementary Material

EXPERIMENT 1: Data Analysis Supplemental Data

Pleasantness

A repeated-measures ANOVA (ANOVA RM) evaluated the effects of touch conditions: [*Session*: 2 (Direct vs. Vicarious)] and [*Velocity*: 4 (AT = 3 and 6 cm/s; NAT = 18 and 30 cm/s) on perceived pleasantness.

A main effect of *Velocity* [$F(1.39, 50.21) = 57.91$, $p < .001$, $\eta^2_p = 0.617$]. A main effect of *Session* was not found [$F(1, 36) = 2.67$, $p = .11$, $\eta^2_p = 0.069$].

The interaction between *Session*Velocity* was significant [$F(2.76, 99.21) = 9.04$, $p < .001$, $\eta^2_p = 0.201$].

Bonferroni post hoc test comparisons between *velocities* revealed that slow AT velocities (3-6 cm/s) were significantly more pleasant than the faster NAT velocities (18-30 cm/s).

Specifically, AT 3 cm/s (M = 6.00, SE = 0.35) was more pleasant than NAT 18 cm/s (M = 3.51, SE = 0.25): [Mean difference = 2.49, SE = 0.33, ($t(36) = 7.56$, $p < .001$)] and NAT 30 cm/s (M = 2.96, SE = 0.24): [Mean difference = 3.04, SE = 0.38, ($t(36) = 8.06$, $p < .001$)].

The difference between AT 6 cm/s and NAT 18 cm/s was also significant [Mean difference = 2.19, SE = 0.28, ($t(36) = 7.76$, $p < .001$)], as was the difference between AT 6 cm/s and NAT 30 cm/s [Mean difference = 2.74, SE = 0.13, ($t(36) = 8.11$, $p < .001$)]. Additionally, NAT 18 cm/s was significantly rated more pleasant than NAT 30 cm/s [Mean difference = 0.55, SE = 0.13, ($t(36) = 4.31$, $p < .001$)].

No significant difference between AT 3 cm/s and 6 cm/s was found ($p = .397$), suggesting that both velocities were perceived as highly pleasant and comparable.

Following the significant interaction between *Session*Velocity,* the Bonferroni post hoc test revealed that perceived pleasantness in direct and vicarious modalities decreased progressively with the increase in velocity. The AT 3 cm/s was rated as the most pleasant

compared to direct and vicarious NAT 18 cm/s [Mean difference = 2.02, SE = 0.34, (t(36) = 5.86, p < .001); Mean difference = 2.66, SE = 0.31, (t(36) = 8.65, p < .001)] and to direct and vicarious NAT 30 cm/s [Mean difference = 2.45, SE = 0.40, (t(36) = 6.13, p < .001); Mean difference = 3.33, SE = 0.35, (t(36) = 9.55, p < .001)].

A similar pattern defines the other CT-optimal velocity AT 6 cm/s compared to direct and vicarious NAT 18 cm/s [Mean difference = 1.80, SE = 0.31, (t(36) = 5.71, p < .001); Mean difference = 2.45, SE = 0.31, (t(36) = 7.96, p < .001)] and to NAT 30 cm/s [Mean difference = 3.12, SE = 0.34, (t(36) = 9.10, p < .001)].

Notably, there was no significant difference between AT 3 cm/s and AT 6 cm/s in direct and vicarious sessions (p =1.00), highlighting a uniformity of pleasantness perception of 3 cm/s and 6 cm/s CT-Optimal velocities, also comparable in the actual and observed mode.

The administration modality affects the qualitative perception of CT-non-optimal velocity stimulations, specifically NAT 18 cm/s and NAT 30 cm/s. NAT 18 cm/s was perceived as more pleasant in the direct condition than the vicarious one sessions [Mean difference = 0.65, SE = 0.15, (t(36) = 4.36, p =.003)], a similarly pattern for NAT 30 cm/s [Mean difference = 0.88, SE = 0.22, (t(36) = 3.94, p =.01)]. These findings suggest that, overall, watching NAT is assumed less pleasant than when it is experienced on one's skin. These data indicate that stroking velocity and administration modality significantly modulate the perception of touch pleasantness. Slow AT velocities were consistently rated as more pleasant across both modalities. On the contrary, NAT velocities showed a different impact on pleasantness depending on the session: the direct touch maintained a relatively constant level of pleasantness.

Pleasantness and Pain Perception

Three repeated-measures ANCOVA (RM ANCOVA) evaluated the probability of the influence of direct or vicarious pain perception on the pleasantness. As the AT pleasantness perception is the main object of the analysis, and the two CT-Optimal cms/s and 6 cms/s

velocities showed significant similarities, for the following analyses, we considered the overall AT (mean of 3 cms/s and 6 cms/s velocities) and NAT (mean of 18 cms/s and 30 cm/s velocities) tactile experience.

A RM ANCOVA within-subjects [*Session*: 2 (Direct vs. Vicarious)] and [*Velocity*: 2 (AT; NAT)] was conducted, adding VP Intensity (VP_INT) representing a measure of empathy to others' pain perception, as the continuous covariate, to evaluate the effects on perceived pleasantness.

A main effect of *Velocity* was found [$F(1, 35) = 14.86$, $p < .001$, $\eta^2_p = 0.298$]. Bonferroni Post hoc test comparison revealed that AT (M=5.85, SE=0.32) was significantly perceived as more pleasant than the NAT (M=3.24, SE=0.24), [Mean difference = 2.61, SE = 0.31, ($t(35) = 8.28$, $p < .001$)].

A main effect of *Session* was not found [$F(1, 35) = 1.97$, $p = .169$, $\eta^2_p = 0.053$].

A significant interaction between *Session*Velocity* emerged [$F(1, 35) = 15.90$, $p < .001$, $\eta^2_p = 0.312$].

The Bonferroni Post hoc test confirmed that pleasantness in both direct and vicarious modalities decreased progressively with an increase in velocity.

The direct AT (M=5.75, SE=0.33) was rated more pleasant than direct NAT (M=3.62, SE=0.28) and vicarious NAT (M=2.86, SE=0.22)

Likewise, vicarious AT (M = 5.96, SE = 0.35) was rated as more pleasant than both direct NAT and vicarious NAT. These data support the superiority of AT pleasantness, which also does not differ between the direct and vicarious administration. On the contrary, NAT pleasantness was greater in the direct session than in the vicarious one. No effect of the interaction between *VP_INT* and *Session* or *Velocity* or among *VP_INT * Session*Velocity* (all $p > .121$) was found.

A RM ANCOVA within-subjects [*Session*: 2 (Direct vs. Vicarious)] and [*Velocity*: 2 (AT; NAT)] was conducted, adding VP_N representing a measure of empathy to others' pain perception, specifically the number of times participants perceived bodily pain while

watching the VPQ videos as the continuous covariate, to evaluate the effects on perceived pleasantness.

A main effect of *Velocity* was found [$F(1, 35) = 11.44$, $p = .002$, $\eta^2_p = 0.246$]. Bonferroni Post hoc test comparison revealed that AT (M=5.85, SE=0.32) was significantly perceived as more pleasant than the NAT (M=3.24, SE=0.24), [Mean difference = 2.61, SE = 0.31, ($t(35) = 8.28$, $p < .001$)].

A main effect of *Session* was not found [$F(1, 35) = 0.62$, $p = .435$, $\eta^2_p = 0.018$].

A significant interaction between *Session*Velocity* emerged [$F(1, 35) = 12.67$, $p = .001$, $\eta^2_p = 0.266$].

The Bonferroni Post hoc test confirmed that pleasantness in both direct and vicarious modalities decreased progressively with an increase in velocity, consistent with the previous RM ANCOVA results.

These RM ANCOVA results suggest that individual sensitivity to vicarious pain perception did not influence the perception of tactile pleasantness.

A last RM ANCOVA within-subjects [*Session*: 2 (Direct vs. Vicarious)] and [*Velocity*: 2 (AT; NAT)] was conducted, adding Direct Pain Perception (DP), as the continuous covariate, to evaluate the effects on perceived pleasantness.

No main effect of *Velocity* or *Session or* interaction effect between *Session*Velocity* was found (all $p > .061$), indicating that velocity had a comparable effect on pleasantness across the two delivery modes. Also, no significant interaction effect between *DP* and *Session* or *Velocity* or among *DP * Session*Velocity* (all $p > .206$) was found.

These results suggest that individual threshold sensitivity to direct painful stimuli does not affect the perception of tactile pleasantness.

<u>Vividness</u>

A repeated-measures ANOVA (ANOVA RM) evaluated the effects of touch condition [*Session*: 2 (Direct vs. Vicarious)] and [*Velocity*: 4 (AT = 3 and 6 cm/s; NAT = 18 and 30 cm/s)] on perceived vividness. Huynh-Feldt corrections were applied because of the violated assumptions of Sphericity (Mauchly's test: velocity factor $p < .001$; session*velocity" $p < .001$).

A main effect of *Session* emerged, [$F(1, 36) = 29.92$, $p < .001$, $\eta^2_p = 0.454$].

A main effect of *Velocity* was not found [$F(1.36, 48.95) = 0.948$, $p = 0.363$, $\eta^2_p = 0.026$].

The interaction between *Session*Velocity* was significant [$F(1.97, 70.79) = 3.31$, $p = .043$, $\eta^2_p = 0.084$].

Bonferroni post hoc test comparisons between *sessions* showed that the direct touch condition (M = 6.64, SE = 0.33) was significantly more vivid than the vicarious (M = 4.61, SE = 0.32) [Mean difference = 2.03, SE = 0.37, ($t(36) = 5.47$, $p < .001$)].

Following the significant interaction between *Session*Velocity,* it was suggested that perceived vividness increases progressively with increasing velocity. The direct NAT at 30 cm/s was rated as the most vivid touch. Else, the Bonferroni post hoc test revealed no significant difference in vividness among all the velocities within the observed or direct sessions (all $p > .05$).

Significant differences were found comparing direct and vicarious modalities: the direct NAT 30 cm/s was rated as the most vivid touch compared to the vicarious NAT 30 cm/s [Mean difference = 2.50, SE = 0.43, ($t(36) = 5.76$, $p < .001$); NAT 18 cm/s [Mean difference = 2.62, SE = 0.42, ($t(36) = 6.24$, $p < .001$); AT 6 cm/s [Mean difference = 2.38, SE = 0.43, ($t(36) = 5.48$, $p < .001$)]; and AT 3 cm/s [Mean difference = 2.47, SE = 0.48, ($t(36) = 5.12$, $p < .001$)]. Similarly, the direct NAT 18 cm/s was rated more vivid compared to the vicarious NAT 18 cm/s [Mean difference = 2.20, SE = 0.43, ($t(36) = 5.16$, $p < .001$); NAT 30 cm/s [Mean difference = 2.08, SE = 0.44, ($t(36) = 4.71$, $p = .001$); AT 3cm/s [Mean difference = 2.04, SE = 0.48, ($t(36) = 4.29$, $p = .004$)]; and AT 6 cm/s [Mean difference = 1.95, SE = 0.44, ($t(36) = 4.28$, $p < .002$)].

An analogous tendency was observed for the AT direct touches compared to the vicarious ones.

Direct AT 3 cm/s resulted more vivid than vicarious AT 3 cm/s [Mean difference = 1.63, SE = 0.41, (t(36) = 3.94, p = .010)]; AT 6 cm/s [Mean difference = 1.54, SE = 0.43, (t(36) = 3.57, p = .029)]; NAT 18 cm/s [Mean difference = 1.78, SE = 0.51, (t(36) = 3.52, p = .033)]; but not compared to vicarious NAT 30 cm/s [Mean difference = 1.66, SE = 0.53, (t(36) = 3.12, p = .098)].

Direct AT 6 cm/s was perceived more vivid than vicarious AT 6 cm/s [Mean difference = 1.78, SE = 0.39, (t(36) = 4.58, p = .001)]; AT 3 cm/s [Mean difference = 1.87, SE = 0.40, (t(36) = 4.71, p = .001)]; NAT 18 cm/s [Mean difference = 2.02, SE = 0.44, (t(36) = 4.65, p = .001)]; NAT 30 cm/s [Mean difference = 1.90, SE = 0.46, (t(36) = 4.17, p = .005)].

In summary, a significant difference in perceived vividness is noted between direct and observed touch. Furthermore, any touch is experienced more vividly when it involves direct skin contact rather than being observed in a video, calling for a central role of skin in social touch.

Vividness and Pain Perception

Three repeated-measures ANCOVA (RM ANCOVA) assessed the effect of direct or vicarious pain perception on the vividness of AT (mean of 3 cms/s and 6 cms/s velocities) and NAT (mean of 18 cms/s and 30 cm/s velocities) tactile experience.

A RM ANCOVA within-subjects [*Session*: 2 (Direct vs. Vicarious)] and [*Velocity*: 2 (AT; NAT)] was conducted, adding VP Intensity (VP_INT), the level of response to others' pain (i.e. vicarious pain) as the continuous covariate, to evaluate the effects on perceived vividness.

A main effect of *Velocity* was found [F(1, 35) = 4.51, p = .041, η^2_p = 0.114], but Bonferroni Post hoc test comparison did not reveal a significant difference in vividness between AT (M=5.54, SE=0.28) and NAT (M=5.72, SE=0.31), (p = .480).

A main effect of *Session* emerged [F(1, 35) = 15.43, $p < .001$, $\eta^2_p = 0.306$]. Bonferroni Post hoc test comparison shows that direct touch (M=6.64, SE=0.33) is more vivid than the vicarious (M=4.61, SE=0.32), [Mean difference = 2.03, SE = 0.37, (t(35) = 5.46, $p < .001$)] (see Figure 5).

A significant interaction between *Velocity* VP_INT* emerged [F(1, 35) = 4.40, p = .043, η^2_p = 0.112].

No significant interaction effect between *Session*Velocity* or *VP_INT* or among *VP_INT * Session*Velocity* (all p > .351) was found.

A RM ANCOVA within-subjects [*Session*: 2 (Direct vs. Vicarious)] and [*Velocity*: 2 (AT; NAT)] was conducted, adding VP_N, which represents the number of times participants perceived bodily pain in vicarious pain experiences.

A main effect of *Session* emerged [F(1, 35) = 16.54, $p < .001$, $\eta^2_p = 0.321$]. Bonferroni Post hoc test comparison confirmed that direct touch is more vivid than the vicarious: [Mean difference = 2.03, SE = 0.37, (t(35) = 5.54, $p < .001$)].

No main effect of *Velocity* was found, neither any interaction effects: *Velocity* Session*; or *Velocity* VP_N*; or *Session* VP_N*; or *Velocity* Session*VP_N* (all p > .148).

Another RM ANCOVA within-subjects analysis [*Session*: 2 (Direct vs. Vicarious)] and [*Velocity*: 2 (AT; NAT)] was conducted, adding Direct Pain Perception (DP) as a continuous covariate, to evaluate the effects on perceived pleasantness.

A main effect of *Session* emerged [F(1, 35) = 14.83, $p < .001$, $\eta^2_p = 0.298$]. Bonferroni Post hoc test comparison confirmed that direct touch is more vivid than the vicarious: [Mean difference = 2.03, SE = 0.37, (t(35) = 5.62, $p < .001$)].

No main effect of *Velocity* was found, nor any interaction effects: *Velocity* Session*; or *Session*DP* or *Velocity*DP*; or *Velocity* Session*DP* (all p > .093).

Pleasantness, Vividness and Cutaneous Allodynia

The unexpected presence of eleven participants presenting mild to moderate cutaneous allodynia led us to investigate its role in the pleasantness and vividness experienced with touch.

A RM ANCOVA within-subjects [*Session*: 2 (Direct vs. Vicarious)] and [*Velocity*: 2 (AT; NAT)] was conducted, adding ASC-12 in daily life (ASC-Daily) as a measure of cutaneous allodynia as the continuous covariate, to evaluate the effects on perceived pleasantness. No interaction effect *Session** ASC-Daily, nor *Velocity** ASC-Daily, nor *Session*Velocity** ASC-Daily was found (all $p > .281$).

Similarly, the A RM ANCOVA within-subjects [*Session*: 2 (Direct vs. Vicarious)] and [*Velocity*: 2 (AT; NAT)] was conducted, ASC-12 in daily life (ASC-Daily) as a measure of cutaneous allodynia as the continuous covariate, to evaluate the effects on perceived vividness. No interaction effect *Session** ASC-Daily, nor *Velocity** ASC-Daily, nor *Session*Velocity** ASC-Daily was found (all $p > .398$).

<u>Interplay between tactile pleasant affectivity and pain perception</u>

Following Holm adjustment across all comparisons, the relationships remained statistically significant (p_Holm < 0.05): AT_DPLS-AT_VPLS (p_Holm < 0.001); BDI-STAY-Y1 (p_Holm < 0.001); BDI-STAY-Y2 (p_Holm = 0.033). Other correlations exhibited unadjusted significance (p_raw value < 0.05) but did not surpass the conservative threshold mandated by the Holm adjustment. Given our sample size, the statistical power to detect moderate correlations ($r \approx 0.30$–0.40) under strict Holm correction control was limited. Consequently, these unadjusted associations should be interpreted not as false positives, but as potential exploratory trends that warrant targeted re-examination in larger cohorts with higher statistical power.

Variable 1	Variable 2	N	r	p-value (Original)	p-value (Holm)	Significant (Holm)
**AT_DPLS**	AT_VPLS	37	0,7756	p < .001	p < .001	Yes
**VP_ INT**	VP_N	37	0,6687	p < .001	p < .001	Yes
**BDI**	STAY Y1	37	0,6199	p < .001	0,003	Yes
**BDI**	STAY Y2	37	0,5361	p < .001	0,040	Yes
Direct Pain	VP_ INT	37	0,1724	0,308	1.000	No
Direct Pain	AT_DPLS	37	0,172755	0,307	1.000	No
Direct Pain	VP_N	37	0,28555	0,087	1.000	No
Direct Pain	AT_DVIV	37	-0,077757	0,647	1.000	No
Direct Pain	ASC-Daily	37	-0,049615	0,771	1.000	No
Direct Pain	STQ	37	0,320092	0,053	1.000	No
Direct Pain	AT_VPLS	37	0,131356	0,438	1.000	No
Direct Pain	AT_VVIV	37	0,264103	0,114	1.000	No
Direct Pain	BDI	37	-0,03886	0,819	1.000	No
Direct Pain	STAY Y1	37	0,104795	0,537	1.000	No
Direct Pain	STAY Y2	37	0,295233	0,076	1.000	No
VP_ INT	AT_DPLS	37	0,173641	0,304	1.000	No
VP_ INT	AT_DVIV	37	0,143775	0,396	1.000	No
VP_ INT	ASC-Daily	37	0,002377	0,989	1.000	No
VP_ INT	STQ	37	0,182998	0,278	1.000	No
VP_ INT	AT_VPLS	37	0,131011	0,440	1.000	No
VP_ INT	AT_VVIV	37	0,373683	0,023	1.000	No
VP_ INT	BDI	37	0,169568	0,316	1.000	No
VP_ INT	STAY Y1	37	0,058649	0,730	1.000	No
VP_ INT	STAY Y2	37	0,280745	0,092	1.000	No
AT_DPLS	VP_N	37	0,156522	0,355	1.000	No
AT_DPLS	AT_DVIV	37	0,196212	0,245	1.000	No
AT_DPLS	ASC-Daily	37	0,068413	0,687	1.000	No
AT_DPLS	STQ	37	-0,326454	0,049	1.000	No
AT_DPLS	AT_VVIV	37	0,349363	0,034	1.000	No
AT_DPLS	BDI	37	-0,206995	0,219	1.000	No
AT_DPLS	STAY Y1	37	-0,149968	0,376	1.000	No
AT_DPLS	STAY Y2	37	0,057441	0,736	1.000	No
VP_N	AT_DVIV	37	-0,102769	0,545	1.000	No
VP_N	ASC-Daily	37	0,014441	0,932	1.000	No
VP_N	STQ	37	-0,03469	0,839	1.000	No
VP_N	AT_VPLS	37	0,07447	0,661	1.000	No
VP_N	AT_VVIV	37	0,220011	0,191	1.000	No
VP_N	BDI	37	0,082135	0,629	1.000	No
VP_N	STAY Y1	37	0,213701	0,204	1.000	No
VP_N	STAY Y2	37	0,335677	0,042	1.000	No
AT_DVIV	ASC-Daily	37	-0,095599	0,574	1.000	No
AT_DVIV	STQ	37	0,098786	0,561	1.000	No
AT_DVIV	AT_VPLS	37	0,071548	0,674	1.000	No
AT_DVIV	AT_VVIV	37	0,379028	0,021	1.000	No
AT_DVIV	BDI	37	0,010098	0,953	1.000	No
AT_DVIV	STAY Y1	37	0,033588	0,844	1.000	No
AT_DVIV	STAY Y2	37	0,236029	0,160	1.000	No
ASC-Daily	STQ	37	0,060522	0,722	1.000	No
ASC-Daily	AT_VPLS	37	0,166098	0,326	1.000	No
ASC-Daily	AT_VVIV	37	-0,037499	0,826	1.000	No
ASC-Daily	BDI	37	0,315101	0,057	1.000	No
ASC-Daily	STAY Y1	37	0,022114	0,897	1.000	No
ASC-Daily	STAY Y2	37	0,242807	0,148	1.000	No
STQ	AT_VPLS	37	-0,336341	0,042	1.000	No
STQ	AT_VVIV	37	-0,02279	0,894	1.000	No
STQ	BDI	37	0,321488	0,052	1.000	No
STQ	STAY Y1	37	0,286842	0,085	1.000	No
STQ	STAY Y2	37	0,206176	0,221	1.000	No
AT_VPLS	AT_VVIV	37	0,363055	0,027	1.000	No
AT_VPLS	BDI	37	-0,274875	0,100	1.000	No
AT_VPLS	STAY Y1	37	-0,243773	0,146	1.000	No
AT_VPLS	STAY Y2	37	0,075357	0,658	1.000	No
AT_VVIV	BDI	37	-0,027135	0,873	1.000	No
AT_VVIV	STAY Y1	37	0,007212	0,966	1.000	No
AT_VVIV	STAY Y2	37	0,238773	0,155	1.000	No
STAY Y1	STAY Y2	37	0,387234	0,018	1.000	No

EXPERIMENT 2: Data Analysis Supplemental Data

Pleasantness

A repeated-measures ANOVA (ANOVA RM) evaluated the effects of touch condition [*Session*: 2 (Direct vs. Vicarious)] and [*Velocity*: 4 (AT = 3 and 6 cm/s; NAT = 18 and 30 cm/s) on perceived pleasantness.

A main effect of *Session* was found [F(1, 36) = 6.75, p = .013, η^2_p = .158]. Post hoc test comparisons indicated a significant difference [Mean difference = 0.34, SE = 0.13, (t(36) = 2.60, p = .013)] (between perceived pleasantness in the Direct touch condition (M = 4.93, SE = 0.31) compared to the vicarious one (M = 4.59, SE = 0.27).

A main effect of *Velocity* also emerged, [F(1.19, 42.95) = 48.14, p < .001, η^2_p = .572].

The interaction between *Session*Velocity* was significant [F(2.02, 72.58) = 5.69, p = .005, η^2_p = .137].

Bonferroni Post hoc test comparisons between *velocities* revealed that slow AT velocities (3-6 cm/s) were perceived as significantly more pleasant than the faster NAT velocities (18-30 cm/s). Precisely, AT 3 cm/s (M = 5.87, SE = 0.33) was more pleasant than NAT 18 cm/s (M = 3.97, SE = 0.31) [Mean difference = 1.90, SE = 0.31, (t(36) = 6.43, p < .001)] and NAT 30 cm/s (M = 3.52, SE = 0.32) [Mean difference= 2.35, SE = 0.32, (t(36) = 7.42, p < .001)].

The difference between AT 6 cm/s (M = 5.66, SE = 0.32) and NAT 18 cm/s was also significant: [Mean difference = 1.69, SE = 0.26, (t(36) = 6.57, p < .001)], as the difference between AT 6 cm/s and NAT 30 cm/s: [Mean difference = 2.14, SE = 0.28, (t(36) = 7.58, p < .001)). NAT 18 cm/s was significantly rated more pleasant than NAT 30 cm/s: [Mean difference = 0.45, SE = 0.08, (t(36) = 5.47, p < .001)]. No significant difference between AT 3 cm/s and 6 cm/s (p = .203), signifying that both CT Optimal velocities were perceived as the most pleasant and comparable.

Bonferroni Post hoc analysis of the *Session*Velocity* interaction revealed a systematic decline in perceived pleasantness with increasing stimulation velocity, observed consistently across direct and vicarious modalities.

Direct AT 3 cm/s were rated as the most pleasant compared to direct and vicarious NAT 18 cm/s [(Mean difference = 1.55, SE = 0.34, (t(36) = 4.49, p = .002)]; [Mean difference = 2.18, SE = 0.35, (t(36) = 6.20, p < .001)] and to direct and vicarious NAT 30 cm/s [Mean difference = 1.92, SE = 0.39, (t(36) = 5.03, p < .001)]; [Mean difference = 2.71, SE = 0.36, (t(36) = 7.65, p < .001)];

Likewise direct AT 6 cm/s compared to direct and vicarious NAT 18 cm/s [Mean difference = 1.37, SE = 0.30, (t(36) = 5.60, p = .001); [Mean difference = 2.00, SE = 0.29, (t(36) = 6.92, p < .001)] and to NAT 30 cm/s [(Mean difference = 1.74, SE = 0.33, (t(36) = 5.19, p < .001)]; [Mean difference = 2.53, SE = 0.30, (t(36) = 8.42, p < .001)].

No significant mean differences were found between Direct and Vicarious AT 3 cm/s; between Direct and Vicarious AT 6 cm/s; between Direct AT 3 cm/s and Vicarious AT 6 cm/s; between Direct AT 6 cm/s and Vicarious AT 3 cm/s (all p =1.00).

These data underscore the consistency of pleasantness perception at CT-optimal velocities of 3 cm/s and 6 cm/s in both direct and vicarious modes.

NAT 18 cm/s and NAT 30 cm/s velocities were also affected by the different modalities of touch administration. NAT 18 cm/s was perceived as pleasant in the same way in the direct and in the vicarious session [Mean difference = 0.63, SE = 0.20, (t(36) = 3.10, p =.104)].

Differently, NAT 30 cm/s was perceived as more pleasant in the direct mode than the vicarious one.

[Mean difference = 0.79, SE = 0.22, (t(36) = 3.65, p =.023)].

Also, the pleasantness of direct NAT 18 cm/s and direct NAT 30 cm/s was significantly different [Mean difference = 0.37, SE = 0.10, (t(36) = 3.64, p = .024)], between direct NAT 18 cm/s and vicarious NAT 30 cm/s was significantly different [Mean difference = 1.16, SE = 0.20, (t(36) = 5.70, p < .001)], also between vicarious NAT 18 cm/s and vicarious NAT 30 cm/s [Mean difference = 0.53, SE = 0.13, (t(36) = 3.99, p =.009)].

The results indicate that both stroking velocity and administration modality have a significant effect on the perception of touch pleasantness. Affective touch (AT) velocities were consistently associated with higher pleasantness ratings across direct and vicarious conditions. In contrast, non-affective touch (NAT) velocities exhibited session-dependent effects: while pleasantness ratings for direct touch remained relatively stable, variations were observed in the vicarious modality.

Pleasantness and Pain Perception

Three repeated-measures ANCOVA (RM ANCOVA) evaluated the probability of the influence of direct or vicarious pain perception on the pleasantness of the overall AT (mean of 3 cms/s and 6 cms/s velocities) and NAT (mean of 18 cms/s and 30 cm/s velocities) tactile experience.

A RM ANCOVA within-subjects [*Session*: 2 (Direct vs. Vicarious)] and [*Velocity*: 2 (AT; NAT)] was conducted, adding VP Intensity (VP_INT), representing a measure of vicarious pain perception as the continuous covariate, to evaluate the effects on perceived pleasantness.

A main effect of *Velocity* was found [$F(1, 35) = 21.57$, $p < .001$, $\eta^2_p = 0.381$]. Bonferroni Post hoc test comparison revealed that AT (M=5.77, SE=0.32) was significantly perceived as more pleasant than the NAT (M=3.75, SE=0.32), [Mean difference = 2.02, SE = 0.28, ($t(35) = 7.21$, $p < .001$)].

A main effect of *Session* was found [$F(1, 35) = 4.59$, $p = .039$, $\eta^2_p = 0.116$]. Bonferroni Post hoc test comparison revealed that direct modality (M=4.93, SE=0.31) was significantly perceived as more pleasant than the vicarious (M=4.59, SE=0.28), [Mean difference = 0.34, SE = 0.13, ($t(35) = 2.57$, $p = .015$)].

A significant interaction between *Session*Velocity* emerged [$F(1, 35) = 4.97$, $p = .032$, $\eta^2_p = 0.124$].

The Bonferroni Post hoc test confirmed that pleasantness in both direct and vicarious modalities decreased progressively with an increase in velocity.

The direct AT (M=5.75, SE=0.34) was rated more pleasant than direct NAT (M=4.10, SE=0.36) and vicarious NAT (M=3.39, SE=0.31)

Likewise, vicarious AT (M = 5.79, SE = 0.33) was rated as more pleasant than both direct NAT and vicarious NAT.

No effect of the interaction between *VP_INT* and *Session* or *Velocity* or among *VP_INT * Session*Velocity* was found (all $p > .252$).

These data support the superiority of AT pleasantness, which also does not differ between the direct and vicarious administration. Instead, NAT pleasantness was greater in the direct session than in the vicarious one.

A RM ANCOVA within-subjects [*Session*: 2 (Direct vs. Vicarious)] and [*Velocity*: 2 (AT; NAT)] was conducted, adding VP_N the number of times participants perceived bodily pain while watching the VPQ videos as the continuous covariate, to evaluate the effects on perceived pleasantness.

A main effect of *Velocity* was found [$F(1, 35) = 20.92$, $p < .001$, $\eta^2_p = 0.374$]. Bonferroni Post hoc test comparison revealed that AT (M=5.77, SE=0.32) was significantly perceived as more pleasant than the NAT (M=3.75, SE=0.32), [Mean difference = 2.02, SE = 0.28, ($t(35) = 7.09$, $p < .001$)].

A main effect of *Session* was also found [$F(1, 35) = 5.03$, $p = .031$, $\eta^2_p = 0.126$]. Bonferroni Post hoc test comparison revealed that direct modality (M=4.93, SE=0.31) was significantly perceived as more pleasant than the vicarious (M=4.59, SE=0.28), [Mean difference = 0.34, SE = 0.13, ($t(35) = 2.578$, $p = .014$)].

A significant interaction between *Session*Velocity* didn't emerged [$F(1, 35) = 1.43$, $p = .240$, $\eta^2_p = 0.039$].

No effect of the interaction between *VP_N* and *Session* or *Velocity* or among *VP_N * Session*Velocity* was found (all $p > .264$).

The results of these two RM ANCOVAs indicate that individual sensitivity to vicarious pain perception did not affect the perception of tactile pleasantness.

A last RM ANCOVA within-subjects [*Session*: 2 (Direct vs. Vicarious)] and [*Velocity*: 2 (AT; NAT)] was conducted, adding Direct Pain Perception (DP), as the continuous covariate, to evaluate the effects on perceived pleasantness.

A main effect of *Velocity* [F(1, 35) = 4.13, p = .005, η^2_p = 0.105]. Bonferroni post hoc test revealed a significant Mean difference [M = 2.02, SE = 0.28, (t(35) = 7.12, p < .001)], between (M=5.77, SE=0.32) and NAT (NAT (M=3.75, SE=0.32).

No main effect of *Session* nor interaction effect between *Session**Velocity*, nor interaction effect *DP*Session, or *Velocity* or among *DP * Session** (all p > .064).

These results suggest that individual direct painful perception does not affect the tactile pleasantness experiences.

<u>Vividness</u>

A repeated-measures ANOVA (ANOVA RM) evaluated the effects of touch condition [*Session*: 2 (Direct vs. Vicarious)] and [*Velocity*: 4 (AT = 3 and 6 cm/s; NAT = 18 and 30 cm/s) on perceived vividness. Huynh-Feldt corrections were applied.

A main effect of *Session* emerged, [F(1, 36) = 22.79, p < .001, η^2_p = 0.388].

A main effect of *Velocity* was not found [F(1.16, 41.86) = 3.01, p = 0.085, η^2_p = 0.077].

The interaction between *Session*Velocity* was significant [F(2.44, 88.02) = 4.54, p = .009, η^2_p = 0.112].

Bonferroni post hoc test comparisons between *sessions* revealed that the direct touch (M = 6.08, SE = 0.38) was significantly more vivid than the vicarious (M = 4.35, SE = 0.34) [Mean difference = 2.03, SE = 0.37, (t(36) = 5.47, p < .001)].

Bonferroni post hoc test of the interaction *Session*Velocity* suggested that the perceived vividness is homogeneous within the admiration modality: no significant differences in vividness among all the velocities within the observed or direct sessions were found (all p > 1.000).

Direct AT 3 cm/s resulted more vivid than vicarious AT 3 cm/s [Mean difference = 1.46, SE = 0.39, (t(36) = 3.70, p = .020)]; AT 6 cm/s [Mean difference = 1.52, SE = 0.38, (t(36) = 4.05, p = .007)]; NAT 18 cm/s [Mean difference = 2.42, SE = 0.48, (t(36) = 5.07, p < .001)]; but not compared to vicarious NAT 30 cm/s [Mean difference = 2.43, SE = 0.53, (t(36) = 4.62, p = .001)].

Direct AT 6 cm/s was perceived more vivid than vicarious AT 6 cm/s [Mean difference = 1.42, SE = 0.35, (t(36) = 4.07, p = .007)]; AT 3 cm/s [Mean difference = 1.36, SE = 0.38, (t(36) = 3.55, p = .030)]; NAT 18 cm/s [Mean difference = 2.32, SE = 0.42, (t(36) = 5.52, p < .001)]; NAT 30 cm/s [Mean difference = 2.33, SE = 0.46, (t(36) = 5.03, p < .001)].

Significant differences emerged across direct and vicarious modalities: the direct NAT 30 cm/s was rated as the most vivid touch compared to the vicarious NAT 30 cm/s [Mean difference = 2.14, SE = 0.43, (t(36) = 4.99, p < .001); NAT 18 cm/s [Mean difference = 2.13, SE = 0.40, (t(36) = 5.3, p < .001); but not compared to AT velocities: AT 6 cm/s [Mean difference = 1.23, SE = 0.55, (t(36) = 2.23, p = .898)]; and AT 3 cm/s [Mean difference = 1.17, SE = 0.60, (t(36) = 1.96, p = 1.000)].

Likewise, the direct NAT 18 cm/s was rated more vivid compared to the vicarious NAT 18 cm/s [Mean difference = 1.91, SE = 0.39, (t(36) = 4.96, p < .001); NAT 30 cm/s [Mean difference = 1.93, SE = 0.42, (t(36) = 4.57, p = .002), but not compared to AT 3cm/s [Mean difference = 0.95, SE = 0.57, (t(36) = 1.67, p = 1.000)]; and AT 6 cm/s [Mean difference = 1.02, SE = 0.53, (t(36) = 1.92, p = 1.000)].

In summary, a significant difference in perceived vividness is observed between direct and observed touch. Furthermore, any touch is experienced more vividly when it involves direct as compared to observed skin contact.

Vividness and Pain Perception

Three repeated-measures ANCOVA (RM ANCOVA) assessed the effect of direct or vicarious pain perception on the vividness of AT (mean of 3 cmss and 6 cm/s velocities) and NAT (mean of 18 cms/s and 30 cm/s velocities) tactile experience.

A RM ANCOVA within-subjects [*Session*: 2 (Direct vs. Vicarious)] and [*Velocity*: 2 (AT; NAT)] was conducted, adding VP Intensity (VP_INT), the vicarious pain perception, as the continuous covariate, to evaluate the effects on perceived vividness.

A main effect of *Session* emerged [$F(1, 35) = 14.128$, $p < .001$, $\eta^2_p = 0.288$]. Bonferroni Post hoc test comparison shows that direct touch (M=6.08, SE=0.38) is more vivid than the vicarious (M=4.35, SE=0.35), [Mean difference = 1.74, SE = 0.37, ($t(35) = 4.71$, $p < .001$)].

A main effect of *Velocity* was not found [$F(1, 35) = 0.167$, $p = .0685$, $\eta^2_p = 0.005$].

No significant interaction between *Session*Velocity*, or *Session* VP_INT*, or *Velocity * VP_INT*, or among *VP_INT * Session*Velocity* emerged (all $p > .145$).

A RM ANCOVA within-subjects [*Session*: 2 (Direct vs. Vicarious)] and [*Velocity*: 2 (AT; NAT)] was conducted, adding VP_N, which represents the number of times participants perceived bodily pain in vicarious pain experiences.

A main effect of *Session* emerged [$F(1, 35) = 13.97$, $p < .001$, $\eta^2_p = 0.285$]. Bonferroni Post hoc test comparison confirmed that direct touch is more vivid than the vicarious: [Mean difference 1.74, SE = 0.37, ($t(35) = 4.73$, $p < .001$)].

No main effect of *Velocity* was found, neither any interaction effects: *Velocity* Session*; or *Velocity* VP_N*; or *Session* VP_N*; or *Velocity* Session*VP_N* (all $p > .142$).

Another RM ANCOVA within-subjects analysis [*Session*: 2 (Direct vs. Vicarious)] and [*Velocity*: 2 (AT; NAT)] was conducted, adding Direct Pain Perception (DP) as a continuous covariate, to evaluate the effects on perceived vividness.

A main effect of *Session* was found [F(1, 35) = 9.46, p = .004, η^2_p = 0.213]. Bonferroni Post hoc test comparison confirmed that direct touch is more vivid than the vicarious: [Mean difference = 1.74, SE = 0.37, (t(35) = 4.83, p < .001)].

No main effect of *Velocity* was found (p = .354).

An interaction effect between *Velocity* Session* emerged [F(1, 35) = 5.83, p = .021, η^2_p = 0.059].

No interaction effect of *Session*DP,* or *Velocity*DP,* or *Velocity* Session*DP* was found (all p > .147).

Bonferroni Post hoc test comparison of the interaction *Velocity* Session* confirmed that direct AT (M=6.26, SE=0.38) is more vivid than vicarious AT (M=4.82, SE=0.36): [Mean difference = 1.44, SE = 0.36, (t(35) = 3.99, p = .002)]; direct NAT (M=5.91, SE=0.46) is more vivid than vicarious NAT (M=3.88, SE=0.39): [Mean difference = 2.03, SE = 0.38, (t(35) = 5.23, p < .001)]; direct AT is more vivid than vicarious NAT [Mean difference = 2.03, SE = 0.38, (t(35) = 5.23, p < .001)].

<u>Pleasantness and Cutaneous Allodynia</u>

We also investigate the role of perceived cutaneous allodynia in the pleasantness and vividness experienced with touch.

A RM ANCOVA within-subjects [*Session*: 2 (Direct vs. Vicarious)] and [*Velocity*: 2 (AT; NAT)] was conducted, adding ASC-12 in daily life (ASC-Daily) as a measure of cutaneous allodynia as the continuous covariate, to evaluate the effects on perceived pleasantness. An interaction effect, *Session* *ASC-Daily in daily life [F(1, 35) = 10.30, p = .003, η^2_p = 0.227]. No other interaction effect of *Velocity** ASC-Daily, nor *Session***Velocity** ASC-Daily was found (all p >.539).

A RM ANCOVA within-subjects [*Session*: 2 (Direct vs. Vicarious)] and [*Velocity*: 2 (AT; NAT)] was conducted, adding ASC-12 during the Pain Attacks (ASC-PA) as a measure of cutaneous

allodynia as the continuous covariate, to evaluate the effects on perceived pleasantness. The analysis

results replicate the previous ANCOVA's one. An interaction effect, *Session**ASC-PA [F(1, 35) = 5.9, p = .028, η^2_p = 0.131]. No other interaction effect of *Velocity** ASC-PA, nor *Session***Velocity** ASC-PA was found (all p >.614).

Interplay between tactile pleasant affectivity and pain perception

Following a Holm adjustment for multiple comparisons, 12 pairwise correlations remained statistically significant. These include: AT_DPLS-AT_VPLS (*p*_Holm < 0.001); AT_VPLS-AT_VVIV (*p*_Holm < 0.001); ASC-Daily-ASC-PA (*p*_Holm < 0.001); STAY-Y1-STAY-Y2 (*p*_Holm < 0.001); BDI-STAY-Y2 (*p*_Holm < 0.001); AT_DPLS-AT_DPVIV (*p*_Holm < 0.001); AT_DPLS-AT_VVIV (*p*_Holm < 0.001); VP_INT-VP_NPain (*p*_Holm < 0.001); STQ-STAY-Y2 (*p*_Holm < 0.001); AT_VPLS-AT_DPVIV (*p*_Holm = 0.006); BDI-STAY-Y1 (*p*_Holm = 0.015); and AT_VVIV-AT_DPVIV (*p*_Holm = 0.039). Of the other pairs of correlations that yielded raw significance (*p*_raw < 0.05), none exceeded the conservative criterion established by the Holm procedure. This loss of statistical significance after adjustment could be primarily attributable to the limited statistical power for detecting moderate correlations in our sample size. Rather than reflecting false positives, these uncorrected associations represent noteworthy exploratory trends that warrant further investigation in larger study populations.

Variable 1	Variable 2	N	r	p-value (Original)	p-value (Holm)	Significant (Holm)
**AT_DPLS**	**AT_VPLS**	**37**	**.865**	**< .001**	**< .001**	**Yes**
**AT_VVIV**	**AT_VPLS**	**37**	**.848**	**< .001**	**< .001**	**Yes**
**ASC-Daily**	**ASC-PA**	**37**	**.844**	**< .001**	**< .001**	**Yes**
**STAY-Y1**	**STAY-Y2**	**37**	**.779**	**< .001**	**< .001**	**Yes**
**BDI**	**STAY-Y2**	**37**	**.767**	**< .001**	**< .001**	**Yes**
**AT_DPLS**	**AT_DPVIV**	**37**	**.761**	**< .001**	**< .001**	**Yes**
**AT_DPLS**	**AT_VVIV**	**37**	**.754**	**< .001**	**< .001**	**Yes**
**VP_INT**	**VP_N**	**37**	**.723**	**< .001**	**< .001**	**Yes**
**STQ**	**STAY-Y2**	**37**	**.721**	**< .001**	**< .001**	**Yes**
**AT_DPVIV**	**AT_VPLS**	**37**	**.600**	**< .001**	**.006**	**Yes**
**BDI**	**STAY-Y1**	**37**	**.571**	**< .001**	**.015**	**Yes**
**AT_VVIV**	**AT_DPVIV**	**37**	**.538**	**< .001**	**.039**	**Yes**
STQ	STAY-Y1	37	.497	.002	.117	No
STQ	ASC-PA	37	.468	.003	.225	No
STQ	BDI	37	.426	.008	.544	No
Direct Pain	STAY-Y2	37	-.411	.011	.718	No
Direct Pain	VP_INT	37	-.285	.087	1.000	No
Direct Pain	AT_DPLS	37	.005	.978	1.000	No
Direct Pain	AT_VVIV	37	.093	.584	1.000	No
Direct Pain	AT_DPVIV	37	-.201	.234	1.000	No
Direct Pain	ASC-Daily	37	.028	.870	1.000	No
Direct Pain	STQ	37	-.206	.222	1.000	No
Direct Pain	AT_VPLS	37	.050	.769	1.000	No
Direct Pain	BDI	37	-.277	.097	1.000	No
Direct Pain	STAY-Y1	37	-.346	.036	1.000	No
Direct Pain	ASC-PA	37	-.009	.960	1.000	No
Direct Pain	VP_N	37	-.156	.355	1.000	No
VP_INT	AT_DPLS	37	.121	.477	1.000	No
VP_INT	AT_VVIV	37	.121	.475	1.000	No
VP_INT	AT_DPVIV	37	.026	.879	1.000	No
VP_INT	ASC-Daily	37	-.053	.757	1.000	No
VP_INT	STQ	37	.267	.110	1.000	No
VP_INT	AT_VPLS	37	.140	.407	1.000	No
VP_INT	BDI	37	.232	.167	1.000	No
VP_INT	STAY-Y1	37	.034	.840	1.000	No
VP_INT	STAY-Y2	37	.265	.113	1.000	No
VP_INT	ASC-PA	37	.066	.699	1.000	No
AT_DPLS	ASC-Daily	37	.288	.084	1.000	No
AT_DPLS	STQ	37	-.300	.072	1.000	No
AT_DPLS	BDI	37	.135	.427	1.000	No
AT_DPLS	STAY-Y1	37	-.123	.468	1.000	No
AT_DPLS	STAY-Y2	37	-.162	.338	1.000	No
AT_DPLS	ASC-PA	37	.132	.436	1.000	No
AT_DPLS	VP_N	37	-.127	.455	1.000	No
AT_VVIV	ASC-Daily	37	-.028	.871	1.000	No
AT_VVIV	STQ	37	-.267	.110	1.000	No
AT_VVIV	BDI	37	.113	.505	1.000	No
AT_VVIV	STAY-Y1	37	-.048	.779	1.000	No
AT_VVIV	STAY-Y2	37	-.187	.269	1.000	No
AT_VVIV	ASC-PA	37	-.163	.334	1.000	No
AT_VVIV	VP_N	37	-.058	.732	1.000	No
AT_DPVIV	ASC-Daily	37	.200	.234	1.000	No
AT_DPVIV	STQ	37	-.184	.277	1.000	No
AT_DPVIV	BDI	37	.273	.102	1.000	No
AT_DPVIV	STAY-Y1	37	.011	.948	1.000	No
AT_DPVIV	STAY-Y2	37	-.063	.713	1.000	No
AT_DPVIV	ASC-PA	37	.190	.261	1.000	No
AT_DPVIV	VP_N	37	-.185	.273	1.000	No
ASC-Daily	STQ	37	.322	.052	1.000	No
ASC-Daily	AT_VPLS	37	.074	.663	1.000	No
ASC-Daily	BDI	37	.273	.102	1.000	No
ASC-Daily	STAY-Y1	37	.232	.167	1.000	No
ASC-Daily	STAY-Y2	37	.274	.101	1.000	No
ASC-Daily	VP_N	37	-.144	.394	1.000	No
STQ	AT_VPLS	37	-.299	.073	1.000	No
STQ	VP_N	37	.321	.052	1.000	No
AT_VPLS	BDI	37	.091	.594	1.000	No
AT_VPLS	STAY-Y1	37	-.206	.221	1.000	No
AT_VPLS	STAY-Y2	37	-.217	.196	1.000	No
AT_VPLS	ASC-PA	37	-.035	.838	1.000	No
AT_VPLS	VP_N	37	-.016	.927	1.000	No
BDI	ASC-PA	37	.270	.106	1.000	No
BDI	VP_N	37	.153	.367	1.000	No
STAY-Y1	ASC-PA	37	.168	.320	1.000	No
STAY-Y1	VP_N	37	.087	.611	1.000	No
STAY-Y2	ASC-PA	37	.305	.067	1.000	No
STAY-Y2	VP_N	37	.268	.109	1.000	No
ASC-PA	VP_N	37	.087	.611	1.000	No
